# Design, biological evaluation, solvatochromic, DFT, and molecular docking studies of new metal complexes derived from a semicarbazone ligand

**DOI:** 10.1038/s41598-025-26629-2

**Published:** 2025-11-29

**Authors:** Magdy A. Ibrahim, A. Taha, Omima M. I. Adly, Shery A. Fahmy, Maged Abdelaziz, Nesma Salah

**Affiliations:** 1 Department of Chemistry, Faculty of Education, Ain Shams University, Roxy, Cairo, Egypt; 2 Department of Chemistry, University of Massachusetts, Amherst, MA USA; 3 Department of Chemistry, Faculty of Science, Zagazig University, Zagazig, Egypt

**Keywords:** Semicarbazone, Dipole moments, DFT level, Biological activity, VEGFR-2, Biochemistry, Cancer, Chemistry, Computational biology and bioinformatics, Drug discovery

## Abstract

The current study describes the synthesis and characterization of novel Ni(II), Co(II), and Fe(III) complexes of the semicarbazone ligand (ACMHCA). Spectroscopic, conductance, elemental, and magnetic studies confirmed octahedral geometries. Solvatochromic fluorescence analyses, supported by DFT/TD-DFT calculations, revealed higher excited-state dipole moments indicating increased polarity upon excitation. Among the complexes, Co(II)-ACMHCA displayed the highest polarizability and hyperpolarizability, suggesting potential in optoelectronics. Biological assays demonstrated notable antimicrobial and anticancer activities, with docking studies confirming strong receptor binding. These findings highlight the therapeutic and functional potential of ACMHCA-based metal complexes.

**Supplementary Information:**

The online version contains supplementary material available at 10.1038/s41598-025-26629-2.

## Introduction

Liver cancer, particularly hepatocellular carcinoma (HCC), remains one of the most prevalent and lethal malignancies worldwide. According to recent epidemiological reports, HCC ranks as the sixth most commonly diagnosed cancer and the third leading cause of cancer-related mortality, with over 800,000 new cases and nearly as many deaths annually^1^. Its high fatality rate stems from late-stage diagnosis, limited treatment options, and frequent resistance to conventional chemotherapeutics, underscoring the urgent need for novel and more effective therapeutic agents^2^. In parallel, the rise of antimicrobial resistance represents a global health crisis, reducing the effectiveness of current antibiotics and driving the search for alternative antimicrobial strategies^3^. The development of multifunctional compounds capable of exerting both anticancer and antimicrobial effects is therefore of significant clinical interest^4^. Schiff base ligands and their transition metal complexes have emerged as promising candidates due to their structural versatility, ability to coordinate multiple donor atoms, and capacity to modulate biological activity through metal complexation^5^. These features not only enhance cytotoxicity toward cancer cells but also impart potent antimicrobial activity, making such complexes attractive dual-purpose agents^6^.

Chromone derivatives are effectively extracted from plants and have significant applications in scientific, medical, and industrial fields^7^. Numerous synthetic and naturally occurring chromones were identified to exhibit various biological activities including antimicrobial^8^, antiviral^9^, anti-inflammatory^10^, anti-biofilm^11^, antioxidant^12^, anticancer^13^, antidiabetic^14^, and neuroprotective^15^. Beyond their biomedical significance, chromones are widely utilized in various technological fields including optical and fluorescent systems, photovoltaic devices, photosensors, and metal ion detection^16,17^. 3-Formylchromones are recognized as crucial scaffolds in inorganic chemistry because of their facility to create Schiff bases, hydrazones, and their metal complexes with variable geometrical structures due to their variable coordination modes, which exhibit a broad spectrum of biological and pharmacological activities^18–20^. Semicarbazones are widely used to create several metal complexes due to their flexible coordination modes with metal ions which are sensitive to the reaction conditions^21,22^. The shift in UV/vis absorption and fluorescence maxima with solvent polarity forms the basis of the solvatochromic approach, which is commonly employed to estimate the ground- and excited-state dipole moments of molecules^23^.

In light of our ongoing interest in solvatochromic metal complexes derived from ligands bearing 2-amino-3-formylchromone and semicarbazone moieties^24–27^, the present study focuses on the synthesis and characterization of novel Ni(II), Co(II), and Fe(III) complexes incorporating a hydrazone-based ligand (ACMHCA)^24,25^. These complexes are investigated for their solvatochromic and photophysical behavior across solvents of varying polarity. The dipole moments corresponding to both ground (µ_g_) and excited (µ_e_) states have been quantitatively evaluated through solvatochromic shift analysis, as well as computationally predicted in the gas phase using DFT methodologies^28^.

The anticancer and antimicrobial activities of the synthesized compounds were evaluated, and the results were correlated with selected chemical descriptors to gain insights into their structure–activity relationships. Additionally, molecular docking, a key tool in computational drug design, was employed to investigate the interaction profiles between the compounds and relevant enzyme targets, thereby supporting the rational design and optimization of biologically active agents^29–31^. In our previous work, we demonstrated the relationship between the binding affinity of metal complexes toward specific protein receptors and their corresponding in vitro biological activities^32–34^. Building on insights from our previous studies, this work presents a comprehensive analysis of binding affinities and interaction profiles using molecular docking to facilitate the rational design and optimization of pharmacologically active compounds, ultimately improving the efficiency and success rate of drug discovery.

## Experimental

### Materials

The hydrazone ligand, abbreviated ACMHCA, was prepared as reported^24,25^. Ni(II), Co(II), and Fe(III) nitrate were employed as Merck products. Et_2_O, DMF, DMSO, THF, CHCl_3_, 1,4-dioxane, benzene, acetone, ethyl acetate (Etac), dichloromethane (DCM), and isopropanol (2-PrOH) are the solvents that are used.

### Measurements

The supplementary material provides a comprehensive list of all the instruments employed in the current study.

### Computational method

*Gaussian 09* software package was applied to accomplish geometry optimizations, as well as to investigate the ground and excited state electronic structures and related structural characteristics of the newly synthesized complexes^35^. At DFT/TD-DFT/GENECP calculations, both the standard 6-311G(d, p) basis set for H, C, N, and O atoms and the Effective Core Potentials (GENECP) at SDD basis set for the metal ion were utilized^36,37^. The present metal complexes were examined using a molecular electrostatic potential map (MEP) and the nonlinear optical (NLO) characteristics.

### Antitumor activity

The cytotoxic activity of ACMHCA and its metal complexes was assessed using the MTT assay against the HepG2 human liver cancer cell line. Cells were obtained from VACSERA Tissue Culture Unit, supplemented with 10% fetal bovine serum (FBS), 1% penicillin-streptomycin, and maintained at 37 °C in a humidified incubator with 5% CO₂. Cells were seeded in 96-well plates at a density of 1 × 10^4^ cells per well and allowed to attach for 24 h. Stock solutions of the test compounds and *cisplatin* (reference drug) were prepared in DMSO (10 mg/mL) and diluted with culture medium to final concentrations ranging from 1 to 100 µg/mL. The final DMSO concentration in all wells was kept below 0.5%. After 48 h incubation with the compounds, 20 µL of MTT solution (5 mg/mL) was added to each well and incubated for 4 h. The medium was then removed and 100 µL of DMSO was added to solubilize the formazan crystals. Absorbance was measured at 570 nm (reference: 630 nm) using a microplate reader. The half-maximal inhibitory concentration (IC_50_) values were calculated using GraphPad Prism^38^.

### Antimicrobial screening

The antimicrobial activity of the synthesized complexes was evaluated using the disc agar diffusion method^39,40^. The compounds were tested against a panel of pathogenic microorganisms, including Gram-positive bacteria (*Staphylococcus aureus* and *Bacillus subtilis*), Gram-negative bacteria (*Escherichia coli* and *Salmonella typhimurium*), and fungal strains (*Aspergillus fumigatus* and *Candida albicans*). Sterile filter paper discs (6 mm diameter) were impregnated with the test compounds at appropriate concentrations and placed onto agar plates previously inoculated with the test organisms. After incubation at 37 °C for 24 h for bacteria and 28 °C for 48 h for fungi, the antimicrobial activity was assessed by measuring the diameter of the inhibition zones (in mm). Standard antibiotics were used as positive controls, while solvent-loaded discs served as negative controls.

### Docking studies

Molecular docking *via* Schrodinger, visualized by Biovia (DS2024 3D) software, seeking modulization of protein-ligand interactions of newly synthesized molecules, is a computational procedure essential for understanding how compounds interact with enzymes, predicting their binding patterns and stability within active sites. This approach helps to visualize the optimal configurations of enzyme-compound complexes by minimizing energy and analyzing binding affinities and the best binding scores, RMSD^29–31^. We have targeted VEGFR-2 enzyme (PDB ID: 1YWN) as a human liver carcinoma HepG2 protein, and utilize its crystallographic structure, to study how our synthesized complexes bind and inhibit the enzyme activity^41^.

### Synthesis of the metal complexes

**Scheme 1:**
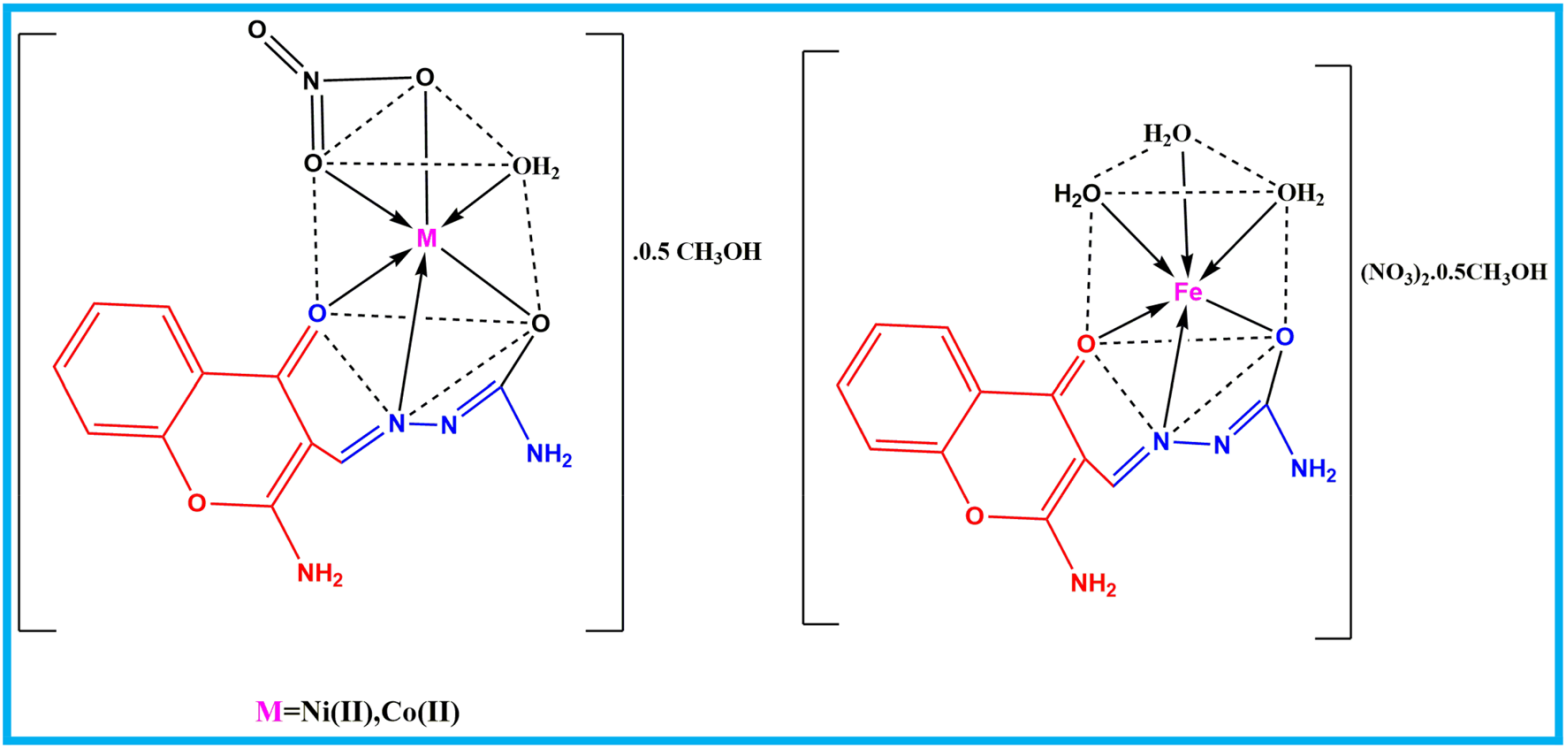
Plausible structure of metal complexes.

A methanolic solution of the ligand (0.74 g, 3.0 mmol in 30 mL) was gradually added to an equimolar (3.0 mmol) of methanolic solution (30 mL) of the metal nitrate in a 1:1 molar ratio; then the mixture was refluxed for 3 h. The resulting solid products were isolated by filtration and subsequently purified through successive washings with diethyl ether and methanol. The purified metal complexes were then stored in a desiccator over anhydrous CaCl_2_ (Scheme 1).

## Structural characterization of the synthesized metal complexes

Based on analytical data, the ACMHCA ligand functions as a monobasic tridentate donor and coordinates with Ni(II), Co(II), and Fe(III) metal ions through ONO donor sites. Physical and analytical data of the resulting complexes are summarized in Table 1. The elemental analysis results of the synthesized complexes closely matched the calculated values, with deviations not exceeding ± 0.4%, which validates the proposed molecular compositions. In addition, molar conductivity measurements in DMF yielded values of 7.50, 16.48, and 163.3 Ω^−1^ cm^2^.mol^−1^ for the Ni(II)-, Co(II)- and Fe(III)-ACMHCA complexes, respectively. These results suggest that the Ni(II)- and Co(II)-ACMHCA complexes behave as non-electrolytes. In contrast, the Fe(III)-ACMHCA complex exhibits the behavior of a 1:2 electrolyte^42^.

**Table 1:** Analytical and physical data of the metal complexes.

No.	Complex	M. F. [F. Wt]	Color	Yield (%)	m.*p*. °C	Elemental analysis, % Found/(Calc.)			
						C	H	N	M
**1**	[(L)Ni(NO_3_)(H_2_O)]0.0.5CH_3_OH	C_11.5_H_13_N_5_O_7.5_Ni [399.96]	Pale Brown	72	> 300	34.53 (34.94)	3.28 (3.80)	17.51 (17.64)	14.67 (14.78)
**2**	[(L)Co(NO_3_)(H_2_O)]0.0.5CH_3_OH	C_11.5_H_13_N_5_O_7.5_Co [400.16]	Brown	67	> 300	34.80 (34.52)	3.11 (3.27)	18.06 (17.50)	14.65 (14.70)
**3**	[(L)Fe(H_2_O)_3_](NO_3_)_2_.0.5CH_3_OH	C_11.5_H_17_N_6_O_12.5_Fe [495.29]	Red	62	> 300	27.96 (27.88)	3.46 (3.45)	16.86 (16.96)	11.52 (11.30)

### Vibrational analysis

**Fig. 1:**
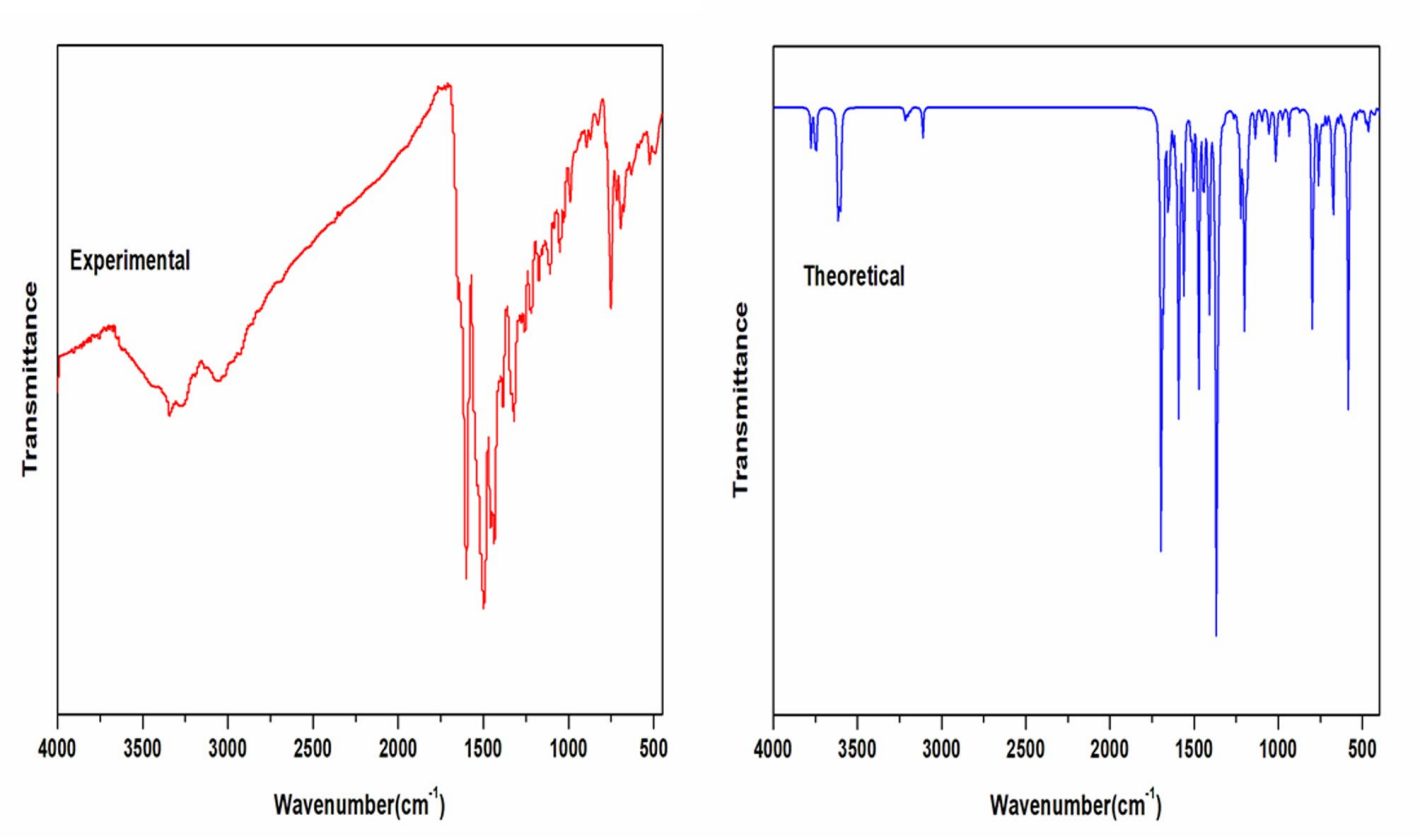
Theoretical and experimental IR spectra of Co(II)-**ACMHCA** complex **2**.

Table 2 presents the characteristic IR absorption bands of the ACMHCA ligand, and its metal chelates, along with their corresponding vibrational mode assignments. The B3LYP/GENECP method at the 6-311G(p, d) level for C, H, N, and O atoms and SDD basis set has been used to predict infrared spectroscopic data of the present complexes. Upon comparing the IR spectra of the synthesized complexes with their free ACMHCA ligand, it was observed a lower shift of the IR bands of the free ligand at 1613 (C = N) and 1659 (C = O_γ−pyrone_) cm^−1^^24,25^ to the ranges 1598–1606 and 1636–1659 cm^1^ upon complexation; suggesting their involvement in the coordination sphere^43^. Figure 1, Figs. S1 and S2 showed the theoretical predictions of C = N and C = O_γ−pyrone_ of the present complexes in the ranges 1608 − 1598 and 1640–1656 cm^− 1^, respectively. The formation of metal-ligand chelation is further supported by the appearance of weak bands at 570–532 and 493–491 cm^− 1^ regions, which can be attributed to M–O and M–N stretching vibrations, respectively^44^. These bands compare very well to the values estimated at 584 − 528 and 488 –464 cm^− 1^ for v(M-O) and ν(M-N), respectively. The NO_3_^−^ ions exhibit bidentate ligand behavior in complexes **1** and **2**. Three non-degenerated vibrational modes (ν, ν_a_, and ν_s_) are present in these nitrate groups, and they were shown at (1496, 1384, 1306, 1079 cm^−1^) and (1484, 1384, 1294, 1105 cm^− 1^), respectively. The calculated bands at B3LYP/GENECP level in the same region show band positions at ranges (1480 –1472 cm^− 1^), (1368 –1320 cm^− 1^) and (1048 –1016 cm^−1^). Whereas Fe(III)-ACMHCA complex **3**, showed two bands at 1384 and 900 cm^− 1^; which agree well with the corresponding theoretical results at 1376 and 944 cm^− 1^, referring to the ionic nature of NO_3_^−^ group^44,45^.

**Table 2:** Characteristic IR spectral data (cm)^−1^ of the ligand and its metal complexes.

Complex		ν(OH)	ν(NH_2_)	ν(C = O) γ-pyrone	ν(C = *N*)	ν(C = C)	ν(M-O)	ν(M-*N*)	Other bands
**1**	[(L)Ni(NO_3_)(H_2_O)]0.0.5CH_3_OH	3405	3261, 3161	1636	1608	1541	570	493	1496, 1384, 1306, 1079; ν(NO_3_^–^) (bidentate)
**2**	[(L)Co(NO_3_)(H_2_O)]0.0.5CH_3_OH	3421	3170	1638	1598	1553	562	492	1484, 1384, 1294, 1105; ν(NO_3_^–^) (bidentate)
**3**	[(L)Fe(H_2_O)_3_](NO_3_)_2_.0.5CH_3_OH	3396	3165	1659	1608	1536	532	491	1384, 900; ν(NO_3_^–^) (ionic)

### Mass spectra

**Fig. 2:**
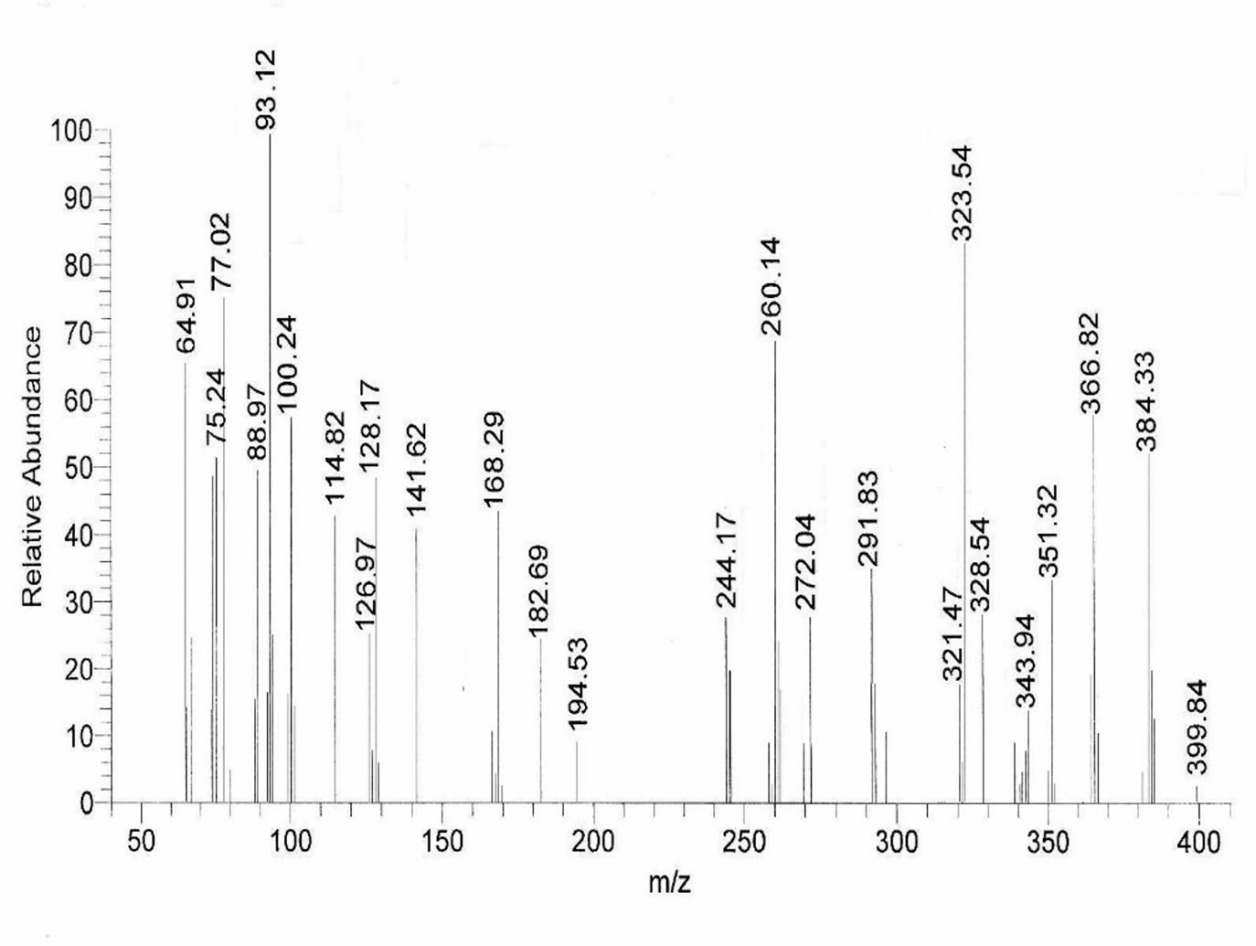
Mass spectrum of Co(II)-**ACMHCA** complex **2**.

**Scheme 2:**
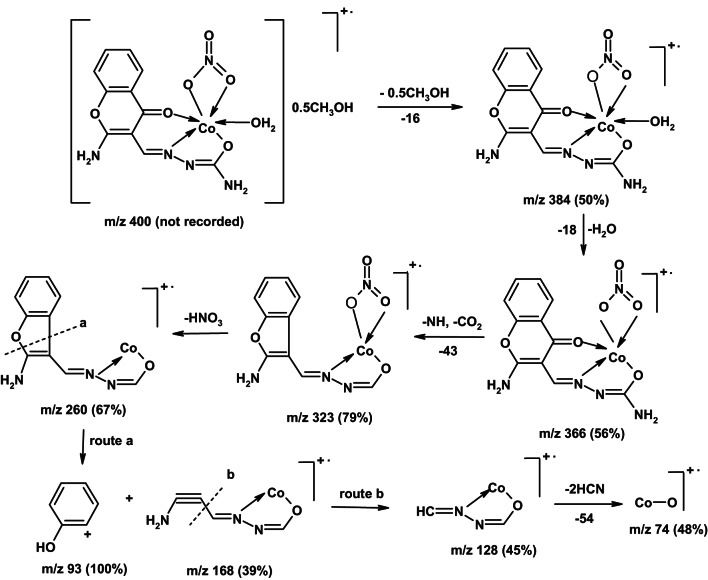
Mass fragmentation patterns of Co(II)-ACMHCA complex **2**.

Figure 2, Figs. S3 and S4 display mass spectral charts of the studied complexes **1**–**3**, molecular ion peaks at *m/z* 385, 384, and 479; supporting their proposed molecular formulas for the anhydrous complexes; [(L)Ni(NO_3_)(H_2_O)] (F.Wt = 383.79), [(L)Co(NO_3_)(H_2_O)] (F.Wt = 384.16) and [(L)Fe(H_2_O)_3_](NO_3_)_2_ (F.Wt = 479.29). Scheme 2 depicts mass fragmentation patterns of complex **2** as an example.

### Electronic spectra and magnetic measurements

As shown in Table 3, the semicarbazone (ACMHCA) ligand and its metal chelates’ electronic spectral data were measured in DMF solution, Nujol mulls, and/or reflectance. The free, ACMHCA, ligand has two electronic transitions at 286 and 316 nm attributed to π-π* and n-π*, respectively^24,25^. In the metal complexes, the π–π* and n–π* electronic transition bands exhibit red shifts of approximately (2–5) nm and (33–37) nm, respectively, relative to those observed for the free ligand^36^.

**Table 3:** Electronic transition bands, magnetic moment (µ_eff_) values and molar conductivity of the metal complexes.

No.	Complex	Electronic spectral bands (nm) λ_max_ (nm) (Nujal mull)	µ_eff_. B.M.	Conductance (Ω^−1^ cm^2^ mol^− 1^)
**1**	[(L)Ni(NO_3_)(H_2_O)]0.0.5CH_3_OH	291, 349, 392, 456, 570 (282, 370, 483, 573)	3.10	7.50
**2**	[(L)Co(NO_3_)(H_2_O)]0.0.5CH_3_OH	288, 353, 390, 444, 534 (286, 346, 540)	4.90	16.48
**3**	[(L)Fe(H_2_O)_3_].(NO_3_)_2_.0.5CH_3_OH	273, 351, 384, 446, 590 (287, 368, 604)	5.82	164.30

The complex of Ni(II)**-ACMHCA** displayed three bands at 392, 456 and 570 nm, which might be attributed to LMCT, ^3^T_1g_(F)←^3^A_2g_ (F) and ^3^T_1g_(P)←^3^A_2g_ (F), respectively, suggesting an octahedral structure^46^. This is confirmed using the magnetic moment value (3.10 B.M.), which falls within the characteristic range (2.9–3.3 B.M.) typically observed for octahedral Ni(II) complexes^47^. However, the ν_1_ band is difficult to observe but can be calculated using Tanab–Sugano diagram and appears at about 941 nm. The calculated ligand field parameters B = 487 cm^−1^, 10Dq = 12,515 cm^−1^, and β = 0.45 fall within the expected range for octahedral complexes, supporting the proposed coordination geometry^48^.

On the other hand, the Co(II)**-ACMHCA** electronic spectrum demonstrated three electronic bands at 390, 444 and 534 nm that are attributed to LMCT, ^4^T_1g_(P)←^4^T_1g_(F), and ^4^A_2g_ (F)←^4^T_1g_ (F), verifying octahedral geometry^49^. The Co(II)**-ACMHCA** magnetic moment is 4.90 B.M, so a high-spin octahedral geometry was suggested^47^. The ν_1_ band was not detected; however, its position was estimated from the corresponding Tanabe–Sugano diagram to occur at approximately 929 nm. The calculated ligand field parameters Racah parameter B (538 cm^− 1^), crystal field splitting energy 10Dq (11,606 cm^−1^), and the nephelauxetic ratio β (0.55) fall within the expected range for complexes exhibiting an octahedral geometry^48^. Finally, the complex Fe(III)**-ACMHCA** presented bands at 384, 446 and 590 nm. However, additional d–d transitions were not resolved due to the presence of an intense charge transfer (CT) band extending from the ultraviolet into the visible region. The Fe(III)-**ACMHCA** complex exhibited a magnetic moment of 5.82 B.M, consistent with a high-spin octahedral configuration^46^. Although the ν₁ transition band was not observed experimentally, it was theoretically estimated at 334 nm. Ligand field analysis yielded the parameters B = 514 cm^−1^, 10Dq = 13,549 cm^− 1^, and a nephelauxetic ratio (β) of 0.46, all of which align well with characteristic values reported for octahedral Fe(III) complexes.

### Thermal analysis (TGA)

**Fig. 3:**
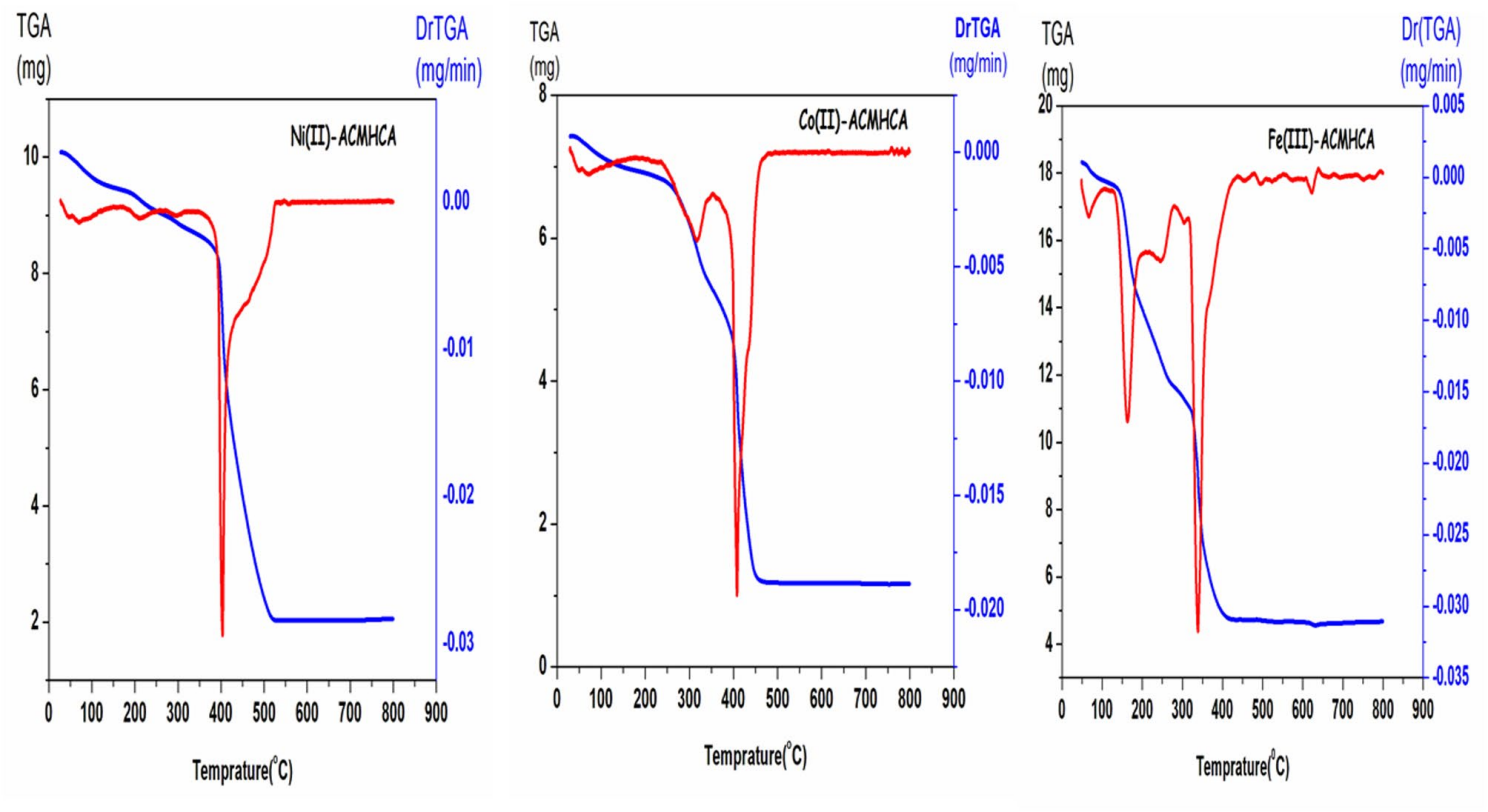
Thermogram of the current complexes.

The TGA and DTG were achieved on the isolated solid complexes **1**–**3** in the temperature range of room temperature up to 800 °C to determine the nature of associated water/methanol molecules as well as their thermal stability (Fig. 3). The thermogram of Ni(II)**-ACMHCA**, [(L)Ni(NO_3_)(H_2_O)]0.0.5CH_3_OH, exhibited three decomposition steps within the range of 27–420 °C as depicted in Table S1. The first, at 27–101 °C, was assigned to the removal of half an uncoordinated methanol molecule. The second stage was observed in the range of 101–232 °C that attributed to the elimination of one chelated water molecule. The third at 232–420 °C, which may be due to the elimination of HNO_3_ and C_11_H_9_N_4_O_2_ molecules. The residue due to the decomposition of the rest of the complex was nickel oxide molecule and carbon atom.

Also, the thermogram of Co(II)**-ACMHCA**, [(L)Co(NO_3_)(H_2_O)]0.0.5CH_3_OH, showed decomposition steps at 33–553 °C. The former at 33–101 °C, which is assigned to the elimination of half uncoordinated methanol molecule. The second, at 101–334 °C and could be attributed to the elimination of one chelated H_2_O molecule and one molecule of HNO_3_. The third at 334–553 °C and may be due to loss of C_11_H_7_N_4_O_2_. The residue due to the decomposition of the rest of the complex was cobalt oxide.

In addition, Fe(III)**-ACMHCA**, [Fe(L)(H_2_O)(EtOH)]NO_3_.2H_2_O, exhibited five decomposition steps at 53–564 °C. The former one at 53–80 °C which may be attributed to the elimination of half of non-chelated methanol molecule. The second, at 80–200 °C and may be attributed to the loss of three chelated H_2_O and two molecules of HCN. The third one at 200–311 °C, which could be attributed to the release of two NH_2_ and an oxygen molecule. The fourth step at 311–356 °C, which may be attributed to the loss of two molecules of HNO_3_. The fifth step at 356–550 °C, which may be due to the loss of one molecule of C_4_H_4_. The residue due to the decomposition of the complex was a FeO molecule and 5 C atoms.

The activation thermodynamic parameters for the decomposition of the complexes were determined through Cost-Redfern equations, and the distinct decomposition steps are systematically summarized in Table 4^50^. Kinetic parameters, particularly the activation energy (E_a_), play a crucial role in evaluating the thermal stability of complexes^51^. The computed activation energies (E_a_) for the first dehydration steps of complexes **1**–**3** were 48.57, 42.99, and 117.42 kJ·mol^−1^, respectively. The stability of the current chelates follows the descending order (based on the E_a_ values): **1** > **2** > **3**. Moreover, the activation energies (E_a_) associated with the second stage of chelates **1** and **2** are less than those observed in the respective first stage, suggesting the decomposition rate in the second step is faster than in the first one^52^. The other kinetic parameters shown in Table 4 include the following points:

**Table 4:** Decomposition temperatures of metal complexes and their kinetic parameters.

Compound	stage	*n* order	T (K)	A (S^− 1^)	Δ E (kJ mol^− 1^)	ΔH (kJ mol^− 1^)	ΔS (kJ mol^1^ K^− 1^)	ΔG (kJ mol^− 1^)
**1**	1st	1	343	8.1179 × 10^6^	48.57	45.72	-0.122	87.64
	2nd	1	457	7.533454 × 10^− 6^	9.11	5.31	-0.259	123.74
	3rd	0.66	697	375.3295	49.09	43.29	-0.211	190.37
**2**	1st	1	346	8.5266 × 10^5^	42.99	40.11	-0.141	88.90
	2nd	0	567	7.9621 × 10^− 5^	25.92	21.20	-0.003	22.79
	3rd	0	685	39.519	34.95	29.26	-0.229	186.54
**3**	1st	1	345	9.565 × 10^17^	117.42	114.55	-0.089	145.48
	2nd	0	441	2.1396 × 10^6^	58.93	55.26	-0.135	114.89
	3rd	1	518	2.0727 × 10^5^	20.90	16.59	-0.156	97.42
	4th	1	616	14418.62	55.98	50.86	-0.156	161.57
	5th	1	723	2.89 × 10^10^	12.06	6.04	-0.052	43.64

1. The positive signs of ΔH* are positive, revealing endothermic behavior of decomposition reactions.
2. The comparatively low positive values of ΔG* suggest a non-spontaneous and autocatalytic effect of the metal ion that facilitates the process^53^.
3. The negative entropy changes (ΔS*) proposed that the reactions are slower than anticipated and the transition state exhibits a higher degree of order compared to the reactants^54^.

## Photophysical properties

### Solvatochromism

The effect of solvents on absorption and fluorescence spectra of the current compounds was investigated in solvents of varying polarities, which were used to estimate the µ_g_ and µ_e_ dipole moments. Table S2 lists the calculated values for various solvent parameters. Further, insights into the solvatochromic characteristics were obtained using multilinear regression analysis of various solvent parameters approaches on the spectroscopic data (Table 5). These data reveal a red shift with increasing solvent polarity, indicative of a π→π^*^ electronic transition^36,55^. The extent of red shifts that reflect the strength of solute-solvent interactions was found in the range of 401–449, 401–443 and 405–455 nm; corresponding to Stoke shifts 2666, 2211 and 2711 cm^−1^, analogous to Ni(II)-, Co(II)- and Fe(III)-**ACMHCA** complexes, respectively^56,57^.

**Table 5:** Excitation and emission data of complexes in diverse solvents at room temperature.

Solvent	Ni(II)-ACMHCA 1 λ_ex_ = 349 nm		Co(II)-ACMHCA 2 λ_ex_ = 343 nm		Fe(III)-ACMHCA 3 λ_ex_ = 351 nm	
	λ_em_	(ν_a_-ν_f_)	λ_em_	ν_a_-ν_f_	λ_em_	ν_a_-ν_f_
1,4-Dioxane	428	5288	409	4704	438	5658
Benzene	401	3715	405	4463	405	3798
Chloroform	435	5664	410	4764	430	5234
Ethyl acetate	425	5123	401	4216	421	4737
Isopropanol	430	5397	443	6166	443	5916
Acetone	444	6130	423	6059	443	5916
Ethanol	439	5874	429	5844	439	5710
Methanol	427	5234	429	5844	445	6018
DMF	449	6381	440	6427	455	6512
THF	436	5717	439	6375	447	6118

### Dipole moment Estimation for the ground and excited States

Variable solvatochromic techniques were employed to calculate the ground and excited-state dipole moments of the presented compounds^27^. Using slopes of the fitted straight lines of Lippert-Mataga, Bakhshiev, and Reichardt (E_T_^N^) *versus* Stokes shift $\left( {\Delta \bar {\upsilon }={{\bar {\upsilon }}_a} - {{\bar {\upsilon }}_f}} \right)$, whereas Kawski-Chamma-Viallet’s *versus* the arithmetic mean of Stokes shift ${{\left( {{{\bar {\upsilon }}_a}+{{\bar {\upsilon }}_f}} \right)} \mathord{\left/ {\vphantom {{\left( {{{\bar {\upsilon }}_a}+{{\bar {\upsilon }}_f}} \right)} 2}} \right. \kern-0pt} 2}$ equations (Figs. S5-S8). The slopes and intercepts are collected in Table S3. The radius, ground, and excited states’ dipole moments are collected in Table 6.

**Table 6:** The dipole moments for the present complexes.

Complex	a/Å	µ_g_^a^	µ_e_^a^	µ_g_^b^	µ_e_^b^	µ_g_^c^	µ_e_^c^	Δµ^d^	Cos φ	φ°
**1**	7.25	11.62	12.28	1.22 (1.64)	10.96 (14.7)	0.62 (0.19)	10.26 (3.08)	10.71 (10.14)	0.89	27.13
**2**	8.10	12.23	13.87	3.22 (3.24)	10.68 (17.6)	0.11 (0.33)	10.28 (3.25)	10.69 (10.70)	0.69	45.65
**3**	8.19	6.64	8.75	6.09 (6.11)	13.49 (13.5)	0.14 (0.16)	12.17 (12.2)	12.29 (12.28)	0.54	56.91

^a^Gaussian 09 calculates ground and excited states using DFT software.

^b^Calculated using (F_1_, F_2_) equations.

^c^Calculated using (F_2_, F_3_) equations.

^d^Calculated from E_T_^N^.

The data in Table 6, clarify that the dipole moment of the present compounds in the excited state is higher than the ground state, suggesting molecule’s excited-state emission is significantly more polar than that of the ground state, likely due to the twisted intramolecular charge transfer (TICT) character of the excited state^58^. Moreover, the π→π^*^ transitions increase in the extension of π^*^ electronic delocalized system and charged resonance structures in the excited state, indicating promising characteristics for applications of the current compounds in nonlinear optical (NLO) materials^59^. The variations of dipole moment values (Table 6) from applying methods arise from the differing theoretical assumptions underlying each method. The limited agreement between experimental solvatochromic shift approaches and DFT theoretical values is comparable to that reported elsewhere^60^. Angles between the ground and excited state dipole moments were calculated, yielding the following values: 27.13°, 45.65° and 56.91° for Ni(II)-, Co(II)- and Fe(III)-**ACMHCA**, respectively. These angles indicate that the ground and excited state dipole momenta aren’t parallel to each other^61^.

This difference might be attributed to some reasons, such as: (i) confidence in Onsager’s theory, which identifies non-specific electrostatic solute-solvent interactions; (ii) disappearance of intermolecular H-bonds; (iii) the suggestion of spherical Onsager radius; (vi) DFT provides dipole moment values only for gas-phase molecules.

### Multiple linear regression analysis

The solvatochromic behavior of the current compounds was quantitatively examined through linear solvation free energy relationships (LSFER), employing a comprehensive set of solvent parameters, such as donor number (DN^n^) and acceptor number (AN^n^), Reichardt’s normalized polarity parameter (E_T_^n^), as well as the solvatochromic parameters π* (dipolarity/polarizability), β (hydrogen-bond accepting ability), and α (hydrogen-bond donating ability). Solvent parameters were used as independent variables *versus* Stoke shift. Multiple linear regressions are used for this purpose, using the Kamlet-Taft Eq^62^:

$${\nu _{{\text{stoke}}}}/{\text{c}}{{\text{m}}^{ - 1}}={\nu _o}+{\text{aA}}+{\text{bB}}+{\text{cC}}+ \cdots$$

Where, ν_Stoke_ equal ν_a_-ν_f_, ν_o_ is the gas phase spectral properties at solvent parameter which equal zero, meanwhile A, B, C,. are solvent parameters, and a-c represent the regression coefficients.

Table S4 summarizes the current LSFER results of ν_stoke_/cm^− 1^ with different solvent conditions. The π → π* transition indicates a significant positive solvatochromic effect, caused by stabilizing the π* -orbitals *via* the polarity and hydrogen bonding characteristics of the solvent^27^. This interpretation is confirmed by the negative slopes of the ν_stoke_/cm^−1^ with the parameters of solvent, which are AN^n^, E_T_^n^, and β. Furthermore, ν_stoke_ has positive slopes with the parameters of DN^n^, α, and π*. This means that the red shift is enhanced by the positive slopes of the specific (DN, α), and π* (non-specific) factors; however, the blue shift is determined by the solvent parameters (AN, and E_T_^n^). Additionally, the data obtained in Table S4 are employed to calculate the relative contribution proportion gathered in Table S5, which offers the following recommendations:

1. Based on the conjugate acid-base theory, the examined compounds exhibit acidic character by the solvent’s Lewis basicity, as indicated by the (+ ve slopes of ν_Stoke_/cm^-1^ vs. DN^n^ and -ve slopes of ν_stoke_/cm^-1^ vs. AN). Consequently, the Lewis acidity of the current molecules’ appeared in this direction: Fe(III)-**ACMHCA 3** > Ni(II)-**ACMHCA 1** > Co(II)-**ACMHCA 2**.
2. Contrary to the red shift, it is overcompensated by β, AN, and ET solvent parameters as indicated from the negative slopes of these parameters *versus* ν_stoke_/cm^−1^.
3. In conclusion, the non-specific π* parameter plays a role in the solvatochromic behavior of the current compounds; Co(II)-**ACMHCA** complex **2** has the highest non-specific contribution, whereas Ni(II)-**ACMHCA** complex **1** has the lowest one (Table 6).

Based on the collected data in Tables S4 and S5, the solvatochromic behavior of the current complexes is mainly controlled by non-specific π* and specific DN and β parameters.

## DFT calculations

### Frontier molecular orbitals (FMOs) analysis

**Fig. 4:**
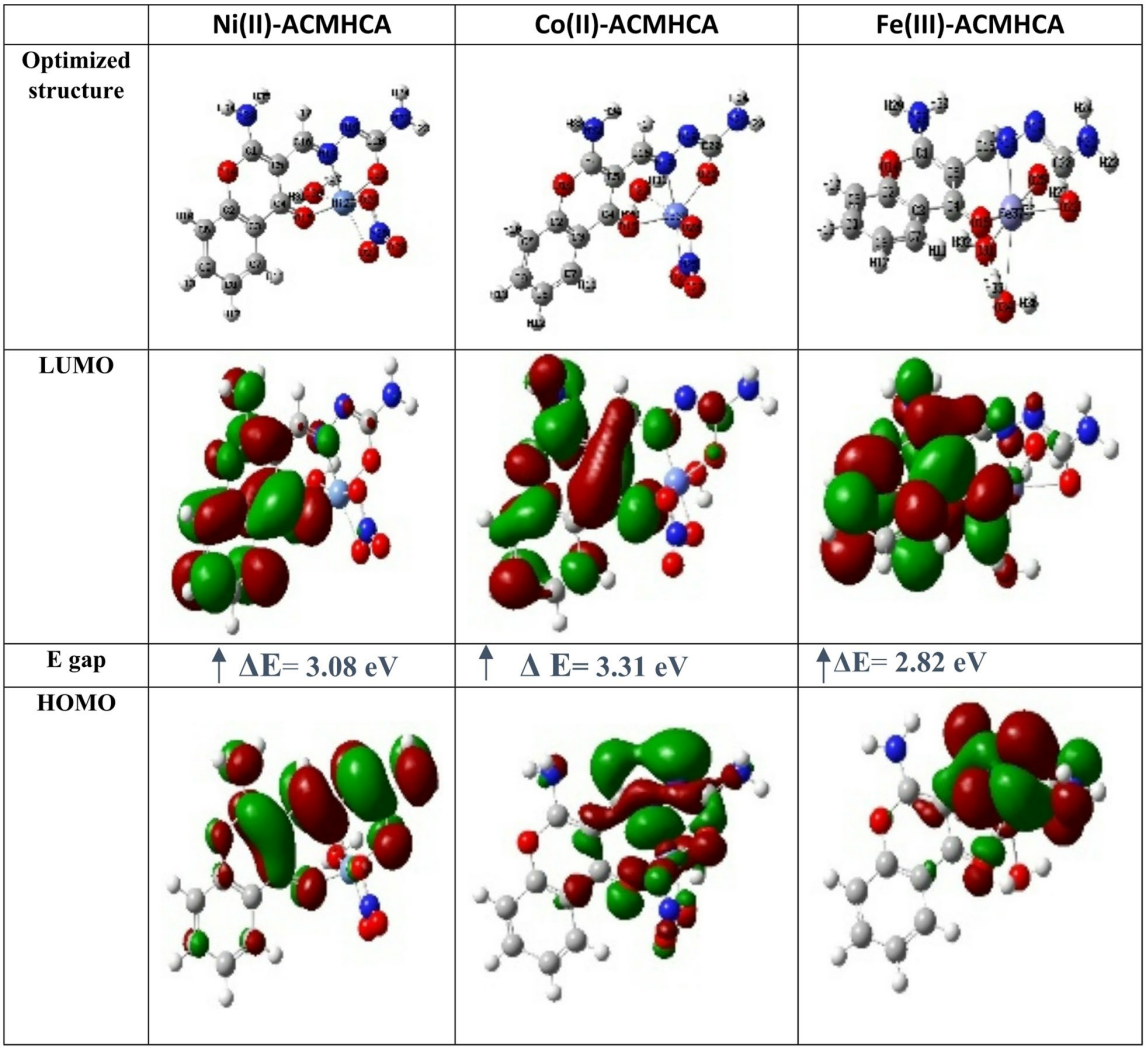
Graphical representation of HOMO-LUMO distribution of metal complexes.

Figure 4 shows the contour shapes of the optimized structures of the synthesized compounds using the DFT/TD-DFT-B3LYP/GEN method in Gaussian 09. The positive and negative regions of the FMOs of LUMO (E_LUMO_) and HOMO (E_HOMO_) in red and green colors, respectively. For the **ACMHCA** ligand, the electron densities in HOMO are concentrated at the side chain and pyrone moiety, while in LUMO are localized at the whole molecule except the amino group. However, metal complexes localized HOMO over the side chain, amino and nitro groups, as well as water molecules, whereas the LUMO is localized on the whole molecule. On the other hand, for Ni(II)-**ACMHCA** complex **1**, the HOMO is concentrated over the pyran moiety, side chain and amino group, but LUMO is localized at the whole molecule except side chain, nitro group and water molecule. However, the HOMO of Co(II)-**ACMHCA** complex **2**, is concentrated over the side chain, amino and nitrate groups and water molecule while the LUMO is localized on the whole molecule except the side chain, nitro group and water molecule. Finally, the HOMO of Fe(III)-**ACMHCA** complex **3**, is localized over the side chain and water molecules, while the LUMO is localized over the whole molecule except the side chain and water molecules. The magnitude of the energy gap between the HOMO and LUMO serves as an indicator of the ease with which electrons can transition from a filled molecular orbital to an unoccupied one^63^. To some extent, the energy gap reflects the ability of the compounds to contribute to chemical reactions.

The extent of the E_gap_ between the HOMO and LUMO is diagrammed in Fig. 4, showing an increase in the following order: 2.82, 3.08 and 3.31 eV for Ni(II)-, Co(II)- and Fe(III)**-ACMHCA** complexes. This order of energy gap reflects the reactivity pathway of optical and chemical performance^64^.

### Quantum chemical reactivity descriptors

Quantum chemical reactivity descriptors of the synthesized molecules collected in Table S6, such as absolute hardness (η), absolute softness (S), electronegativity (χ), chemical potential (Pi) and global electrophilicity (ω); were derived from E_HOMO_’s and E_LUMO_’s^65^. These descriptors indicate that Fe(III)-**ACMHCA** complex **3** has the highest softness value (0.71 eV*)* and lowest absolute hardness (1.41 eV), confirming that this complex is the softness molecule. Ni(II)-**ACMHCA** complex **1** has the lowest softness (0.55 eV) and highest hardness (1.81 eV). Hence, complex **3** is the most reactive one^66^. The calculated electronegativities (χ, eV) show that Fe(III)-**ACMHCA** complex has the lowest value, consistent with its cationic nature and reduced ability to attract electrons. In contrast, Co(II)-**ACMHCA** (**2**) exhibits higher electronegativity, reflecting its stronger electron-withdrawing tendency. As summarized in Table S6, the variation in electronegativity is strongly linked to chemical susceptibility: lower values correspond to higher reactivity and lower stability, while higher values indicate greater resistance to electronic perturbations. This highlights electronegativity as a key predictor of the reactivity and stability of the complexes.

However, the order of global electrophilicity (ω) values is free ligand **ACMHCA**, < Ni(II)-**ACMHCA** (**1)** < Co(II)-**ACMHCA** (**2**) < Fe(III)-**ACMHCA** (**3**). Hence, Fe(III)-**ACMHCA** complex **3** has the highest tendency of electron acceptors to acquire additional electronic charge from the environment^67^.

The dipole moment values are 5.48, 11.62, 12.23 and 6.65 Debye for **ACMHCA** ligand, Ni(II)-**ACMHCA** complex **1**, Co(II)-**ACMHCA** complex **2** and Fe(III)-**ACMHCA** complex **3**, respectively. An increasing dipole moment value might enhance the reactivity of the compound^68^.

Collaborations of the computed quantum chemical descriptors in Table S6 vs. experimental data, supported the current assignments of the experimental data such as:

i. The linearity of E_HOMO_, which measures the reactivity (instability) of the complex vs. the IR data reveals, E_HOMO_/eV = -4.915 – Δν_C=N_/cm^−1^, *r* = 0.88 (*n* = 3 points) (Fig. S9); the -ve slope specifies a decrease of E_HOMO_ with the extent of shifting of C = N to a lower frequency.
ii. Additional proof of the connection between experimental and theoretical information, the dipole moment (µ) vs. IR data, µ/D = -286.55 + 0.182 ν_C=N_/cm^−1^, *r* = 0.99 (*n* = 3 points) (Fig. S10), the + ve slope, refers to an increase of the C = N stretching frequency, which was accompanied by an increase in the polarity of the complex.

Moreover, in all metal complexes, the length of bonds of **ACMHCA** in the vicinity of coordinating sites is typically longer in the following ranges: C_23_-O_27_ (0.049–0.095) and C_4_ = O_14_ (0.019–0.038) A° as shown in Table S7. Additionally, the bond length of C15 = N21 of the free ligand was elongated upon complexation, supporting involvement as a coordination center (Table 2). This finding further confirmed the following relationships: ν_M-O_/cm^-1^ = 731.3– 82.56 _M-O/A°_, *r* = 0.89 (*n* = 3 points) and ν_M-N_/cm^-1^ = 508.81–7.91 _M-N/A°_, *r* = 0.99 (*n* = 3 points). The -ve slopes imply an increase in bond strength. However, the partial negative charge that has collected on the O(14), O(27), and N(21) (Table S7) indicates that these sites are involved in coordination, which is consistent with the above-mentioned IR findings.

### Molecular electrostatic potential (MEP)

**Fig. 5:**
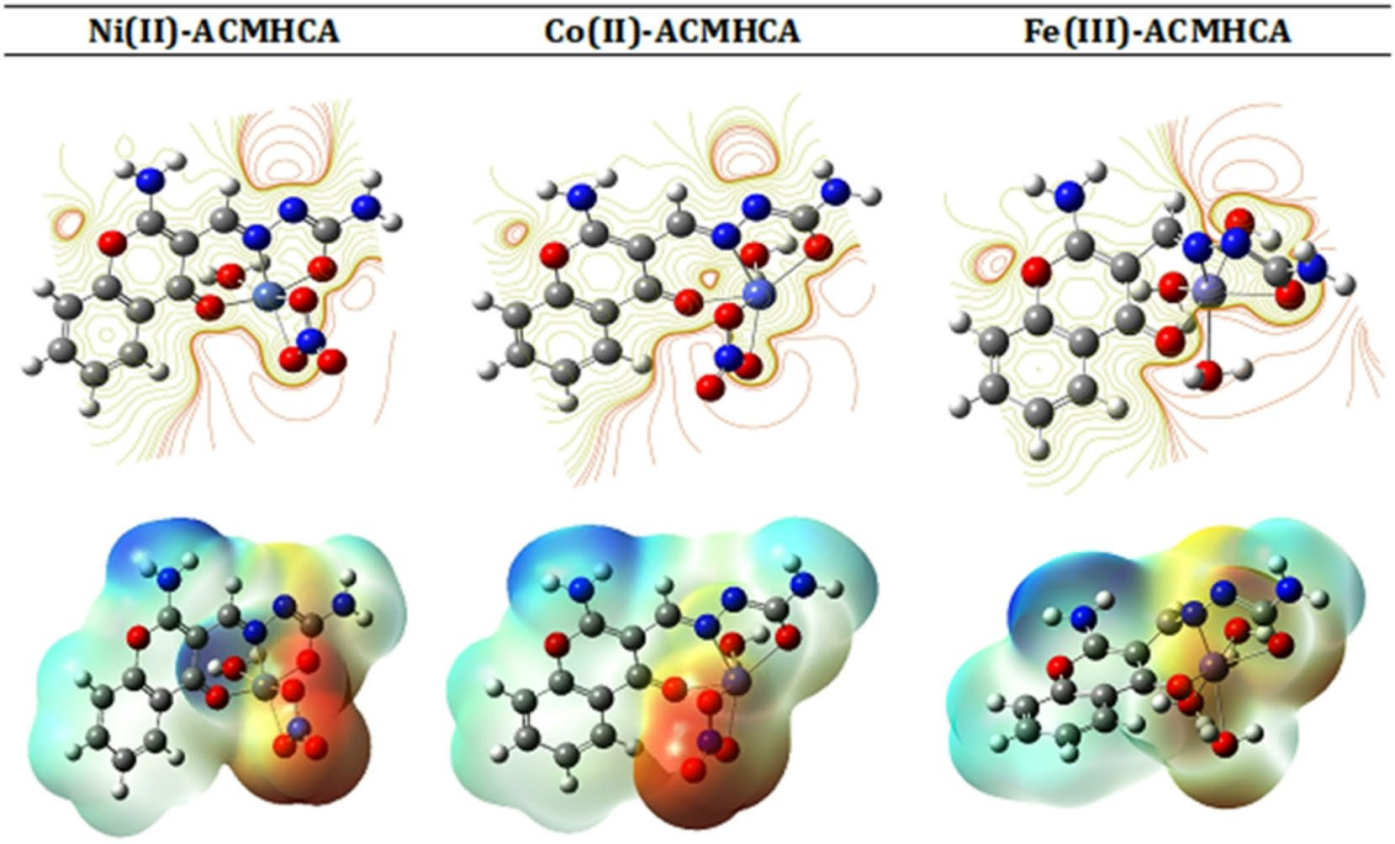
MEP diagram from contour isosurface density and Alpha SCF density of complexes.

MEP represents the electric field potential energy both within and surrounding a molecule due to charge distribution; it affects the structure and characteristics within the molecule and influences interactions with other molecules^69^. Figure 5 depicts the MEP diagram of the current compounds, where the colors gradually shift from red (lowest electrostatic potential), to orange, yellow, green, and finally blue (highest electrostatic potential). For the **ACMHCA** ligand, it was observed that the highest electronegative area exists around the oxygen atoms of C = O_γ-pyrone_ and enolate groups, as well as the nitrogen atom of C = N_azomethine,_ confirming that these sites have precise donor characteristics. Additionally, the compound’s hydrogen atoms are surrounded by positively charged areas. However, the MEP of metal complexes showed the highest electronegative area over the γ-pyrone ring oxygen, azomethine nitrogen, enolate oxygen, nitrate group, as well as water molecules, which agrees well with the HOMO and LUMO contours mentioned above.

### Non-linear optical (NLO) activity

Nonlinear optical efficiency arises from the intense electric polarization, which enhances molecular processes and strengthens the relationship between structure and properties^70^. Tables S8 and S9 summarize NLO descriptors estimated based on DFT methods (Eqs. 1–4)^71^.

1 $$\mu ={\left( {\mu _{x}^{2}+\mu _{y}^{2}+\mu _{z}^{2}} \right)^{1/2}}$$

2 $$\alpha ={{\left( {{\alpha _{xx}}+{\alpha _{yy}}+{\alpha _{zz}}} \right)} \mathord{\left/ {\vphantom {{\left( {{\alpha _{xx}}+{\alpha _{yy}}+{\alpha _{zz}}} \right)} 3}} \right. \kern-0pt} 3}$$

3 $$\Delta \alpha ={(2)^{ - 0.5}}{\left[ \begin{gathered} {\left( {{\alpha _{xx}} - {\alpha _{yy}}} \right)^2}+{\left( {{\alpha _{yy}} - {\alpha _{zz}}} \right)^2}{\left( {{\alpha _{zz}} - {\alpha _{xx}}} \right)^2} \hfill \\ +6{\left( {{\alpha _{yz}}} \right)^2}+6{\left( {{\alpha _{xy}}} \right)^2}+6{\left( {{\alpha _{xz}}} \right)^2} \hfill \\ \end{gathered} \right]^{0.5}}$$

4 $${\beta _{tot}}={\left[ {{{\left( {{\alpha _{xxx}}+{\alpha _{xyy}}+{\alpha _{xzz}}} \right)}^2}+{{\left( {{\alpha _{yyy}}+{\alpha _{yzz}}+{\alpha _{yxx}}} \right)}^2}+{{\left( {{\alpha _{zzz}}+{\alpha _{zxx}}+{\alpha _{zyy}}} \right)}^2}} \right]^{0.5}}$$

Where *α* denotes the mean polarizability; *Δα* the anisotropy of the polarizability; and *β* the mean first-order hyperpolarizability, related to the molecule.

There is a strong correlation between a molecule’s nonlinear optical (NLO) properties and its dipole moment, as both are influenced by charge separation and the movement of the electron cloud in response to external electric fields. Based on the calculated data, the dipole moments of the synthesized complexes follow the order: Co(II)-**ACMHCA** > Ni(II)-**ACMHCA** > Fe(III)-**ACMHCA**. Accordingly, both polarizability (*Δα*) and total hyperpolarizability (*β*_*tot*_) follow the same trend. Additionally, the dipole moments of all the studied compounds (Table S8) exceed that of the standard reference molecule urea (*µ*_urea_ = 1.3732 D), suggesting strong potential for intermolecular interactions^70^. The relatively high *β* values (ranging from 0.742 to 2.19 × 10^-30^ esu) are attributed to efficient intramolecular charge transfer (ICT) through π-conjugated systems, supporting the classification of these compounds as NLO-active materials. Notably, the Co(II)-**ACMHCA** complex (complex **2**) exhibited the highest NLO response, with a β value of 2.19 × 10^-30^ esu, approximately six times greater than that of urea (0.3728 × 10⁻³⁰ esu)^70^.

In addition, comparison with other reported NLO materials indicates that our synthesized complexes exhibit moderate activity. For instance, while *p*-nitroaniline (β ≈ 6.8 × 10⁻³⁰ esu) and organometallic systems such as Cu(II), Ni(II), and Fe(III) Schiff base complexes (*β* ≈ 100–1000 × 10⁻^30^ esu) display higher responses, the present complexes show comparatively lower *β* values. This places them above simple reference molecules like urea, yet within the lower range of advanced organic and organometallic NLO materials. Such positioning underscores their promise as NLO-active species, while also suggesting that structural modifications (e.g., incorporation of stronger push–pull substituents) could substantially enhance their NLO performance.

## Biological activity

### Antitumor investigation

**Fig. 6:**
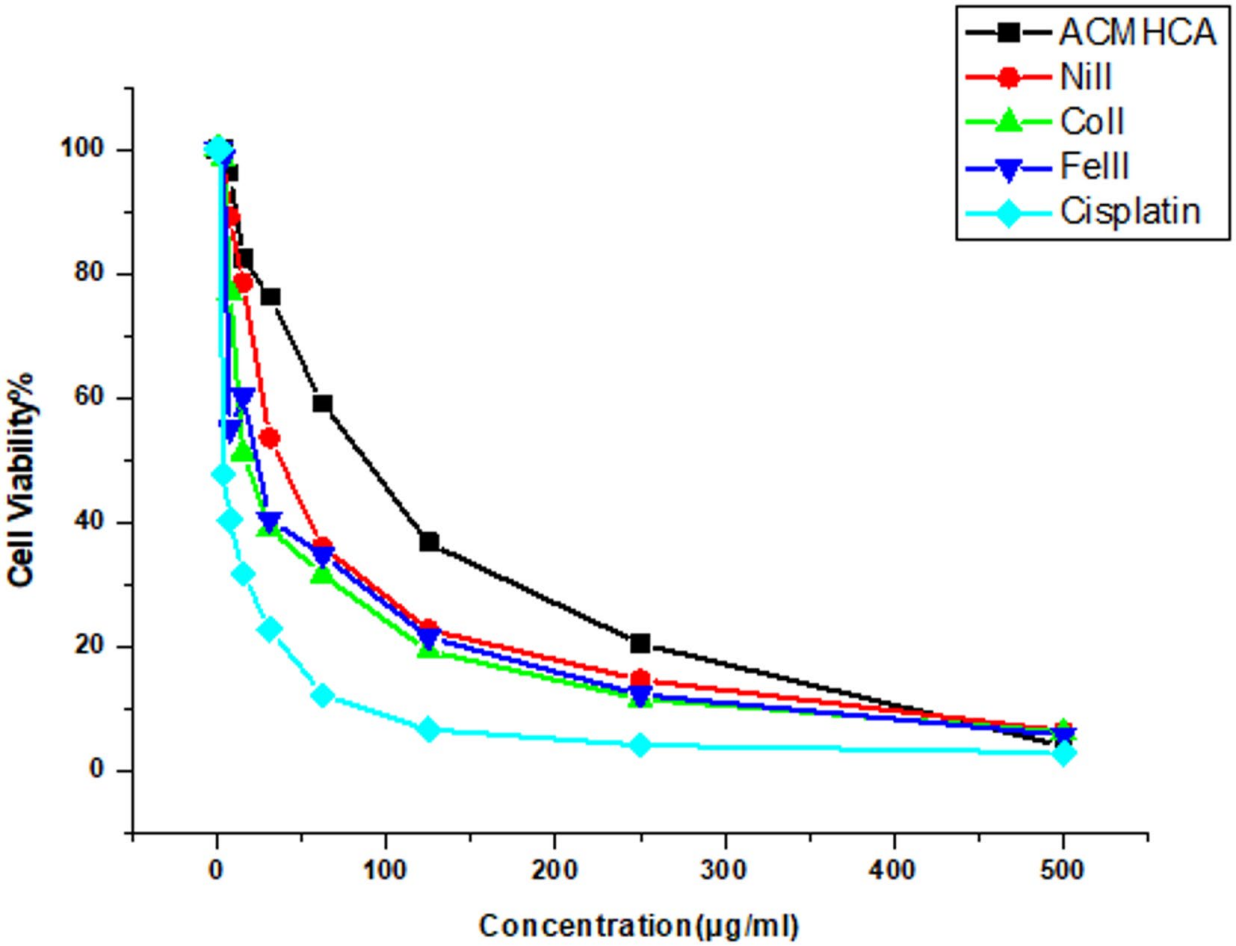
The influence of concentrations of **ACMHCA** and its complex on the growth of the HepG2 cell line.

One of the main objectives of this study was to evaluate the antitumor activity of the **ACMHCA** ligand and its metal complexes against the HepG2 human hepatocellular carcinoma cell line, using *cisplatin* as a reference drug. The data were expressed as IC₅₀ values, the concentration required to inhibit cell viability by 50% (Table 7; Fig. 6).

**Table 7:** Antitumor efficiency of **ACMHCA** and its complexes.

Candidate	IC_50_
**ACMHCA**	49.17 ± 3.62
**1**	26.89 ± 2.56
**2**	14.62 ± 0.98
**3**	5.79 ± 0.96
*Cis-platin*	3.58 ± 0.26

**Fig. 7:**
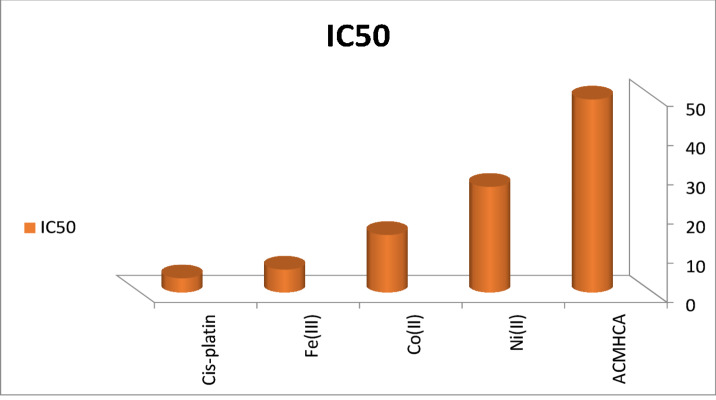
IC_50_ values of the current candidates and *cis-platin* towards HepG2 cell line.

The results showed that metal complexation significantly improved the anticancer activity of **ACMHCA** (Fig. 7). The observed order of potency (from highest to lowest activity) was Fe(III)-**ACMHCA** > > Co(II)-**ACMHCA** > > Ni(II)-**ACMHCA** > > free **ACMHCA**. The free **ACMHCA** ligand and Ni(II) complex showed weak cytotoxicity, with IC₅₀ values of 49.17 and 26.89 µg/mL, respectively. Moreover, The Co(II) complex exhibited moderate activity (IC_50_ = 14.62 µg/mL). The Fe(III) complex displayed the highest activity (IC_50_ = 5.79 µg/mL), which is close to that of cisplatin (3.58 µg/mL). This enhancement is likely due to improved lipophilicity and cell permeability upon complexation, consistent with Overtone’s concept and Tweedy’s chelation theory. The effect of lipophilicity is further supported by computational results discussed below. On the other hand, to understand the structure–activity relationship, the IC₅₀ values were converted to pIC_50_ = log(1/IC_50_) and correlated with DFT-based descriptors. The primary conclusions drawn from the linear regression of pIC_50_ against structural traits and reactivity descriptors are as follows:

i. The E_LUMO_ measures the electrophilicity of a compound. The relation of E_LUMO_ *against* pIC_50_ was obtained as: pIC_50_ = -14.132–6.45 E_LUMO/eV_, *r* = 0.83, *n* = 3 points, a high negative slope of E_LUMO_ with pIC_50_. This indicates that higher E_LUMO_ values (i.e., more stable complexes) are associated with lower cytotoxicity (Fig. S11).
ii. Moreover, the stability of complexes is measured by their hardness (η/eV), pIC_50_ = 2.14–1.66 η/eV, *r* = 0.99, *n* = 3 points) Fig. S12). Lower hardness (more reactive complexes) appears to favor better anticancer activity. This is further supported by the positive slope of softness that shows the reactivity of complex: pIC_50_ = -3.15 + 4.155 S/eV, *r* = 0.99, *n* = 3 points (Fig. S13). More chemically soft (reactive) complexes show improved activity.
iii. However, the dipole moment slightly influences inhibitory activity as shown from the negative slope with a small value: pIC_50_ = 0.412–0.096 µ/D, *r =* 0.88, *n* = 3 points (Fig. S14).

Finally, the results clearly demonstrate that metal coordination enhances the anticancer efficacy of **ACMHCA**, with Fe(III)-**ACMHCA** showing the most promising activity. DFT-based descriptors such as E_LUMO_, hardness, and softness correlate well with biological activity, supporting the experimental findings and providing insight into the structure–activity relationship.

### Antimicrobial evaluations

The antimicrobial efficiency of **ACMHCA** and its metal chelates was investigated against certain microbes that include Gram-positive bacteria (*S. aureus* and *B. subtilis*), Gram-negative bacteria (*E. coli* and *S. typhimurium*), yeast (*C. albicans*), and fungus (*A. fumigatus*); and the results are collected in Table 8. The results demonstrated that the **ACMHCA** exhibited less efficiency than its metal complexes^24,25^. This could be clarified based on electron delocalization across the entire chelate as a result of the positive charge of the metal ion^72^.

**Table 8:** Antimicrobial efficiency of the ligand (**ACMHCA**) and its metal complexes.

Organism	Mean of zone diameter, nearest whole mm.											
	Gram - positive bacteria				Gram - negative bacteria				Yeasts and Fungi			
	*S. aureus*		*B. subtilis*		*S. typhimurium*		*E. coli*		*C. albicans*		*A. fumigatus*	
Sample	Concentration											
	1 mg/ml	0.5 mg/ml	1 mg/ml	0.5 mg/ml	1 mg/ml	0.5 mg/ml	1 mg/ml	0.5 mg/ml	1 mg/ml	0.5 mg/ml	1 mg/ml	0.5 mg/ml
**ACMHCA**	6 L	3 L	5 L	2 L	4 L	2 L	4 L	-	7 L	3 L	5 L	3 L
**1**	24 H	18 H	21 I	15 I	17 I	15 I	9 L	5 L	23 H	19 H	26 H	18 H
**2**	17 I	10 I	16 I	11 I	20 I	12 I	10 L	7 L	25 H	22 H	29 H	20 H
**3**	22 I	17 I	18 I	13 I	13 I	14 I	16 I	11 I	28 H	20 H	30 H	22 H
Control	35	26	35	25	36	28	38	27	35	28	37	26

Control: Chloramphenicol for Gram + bacteria, Cephalothin for Gram - bacteria, cycloheximide for yeast and fungi.

According to the data in Table 8, all complexes have moderate efficiency against *S. aureus* and *S. typhimurium*, except Ni(II)-**ACMHCA**, which exhibits considerable activity. However, all metal complexes have low activities towards *E. coli* and high activities against *Candida albicans* and *A. fumigatus*, except Fe(III)-**ACMHCA** complex **3**, which has intermediate activity.

According to Tweedy’s chelation theory, the efficiency of free **ACMHCA** ligand was significantly enhanced by complexation because (i) the presence of azomethine coordinating centers may promote the creation of H-bonds with the active center of cell components, interfering with the regular cell functions. (ii) Chelation will reduce the polarity of the metal ion *via* delocalization of π-electrons and increase the complexes’ lipophilicity, so it is easier to pass through lipid membranes. (iii) antibacterial efficacy increased when their stability increased. This was supported by the + ve slopes of ν_C=N_ vs. biological activity of *G-1*, Δν_C=N_ as *G-1* = 0.3475 + 0.0135 Δν_C=N_/cm^− 1^, *r* = 0.89, *n* = 3 points. This finding indicates increased biological efficiency accompanied by a higher red shift of ν_C=N_ (more binding interaction).

This evidence is confirmed by the positive correlation of G-1 with the dipole moment, expressed as *G-1* = 0.16053 + 0.029 µ/D (*r* = 0.94, *n* = 3). Additionally, the negative slope of the correlation between E_LUMO_ *vs. G-2* (*G-2* = − 4.469–2.269 E_LUMO_/eV, *r* = 0.98, *n* = 3 points) supports this trend. This is further confirmed by the positive slope obtained for E_HOMO_ *vs. G-2* (*G-2* = 2.29 + 0.385 E_HOMO_/eV, *r* = 0.99, *n* = 3). These results indicate that biological efficiency improves with decreasing complex stability (higher E_HOMO_), which agrees with the behavior observed in the antitumor study.

## Molecular docking studies

A reliable computational approach can be employed to predict the interactions between synthetic candidates and enzyme targets^29–31^. This approach enables detailed exploration of optimal molecular conformations and binding affinities, thereby supporting the rational design of novel chelating agents with minimal or near-zero binding energies. In the present study, in silico molecular docking was performed to evaluate the binding interactions of the ligand (**ACMHCA**) and its metal complexes with the human liver carcinoma receptor (HepG2, PDB ID: 1YWN; 4-amino-furo[2,3-*d*]pyrimidine).

The 1YWN protein was selected as the docking target because it represents a crystallized protein structure relevant to hepatocellular carcinoma, the specific cancer type investigated in this study. HepG2 is a well-established human liver cancer cell line widely used for in vitro anticancer screening, and employing its associated receptor provides a reliable molecular model to evaluate potential binding interactions. Using this target allowed us to directly correlate the computational docking results with the experimental cytotoxicity assays performed on HepG2 cells, thereby strengthening the translational relevance of our findings.

To validate the docking protocol, the co-crystallized ligand of protein 1YWN was re-docked into the main binding pocket, yielding a binding affinity score of − 6.96 kcal/mol. The interactions involved five key amino acid residues: GLU883 (–4.9 kcal/mol), ASP1044 (–2.4 kcal/mol), GLU915 (–3.3 kcal/mol), VAL846 (–0.6 kcal/mol), and CYS917 (–5.0 kcal/mol). These results confirm the reliability of the docking approach and provide a benchmark for evaluating the binding affinities of the synthesized complexes.

The **ACMHCA** ligand was docked with the 1YWN protein enzyme and demonstrated low binding affinity, with a docking score of -5.45 kcal/mol through interactions with the GLU883 amino acid residue in the primary binding pocket with a docking score of -5.45 kcal/mol, the interactions occurred at distances of 2.87 Å and 3.16 Å, with respective interaction energies of -7.0 kcal/mol and − 1.8 kcal/mol. Further, docking studies were conducted for *Cis-platin*, a commercially recognized VEGFR-2 inhibitor; *Cis*-platin stabilized within the same binding pocket, achieving a docking score of -4.19 kcal/mol by interacting with two sites on the ASP1044 residue at distances of 3.92 Å and 3.04 Å, with corresponding interaction energies of -0.7 kcal/mol and − 1.6 kcal/mol. Additionally, all tested compounds exhibited enhanced stability with improved binding values, compared to the binding affinity of the co-crystallized ligand *Cis*-platin in the largest pocket of the 1YWN protein. The results, Table 9, S10 and S11, revealed several key findings:

**Table 9:** Comparative analysis of 2D and 3D receptor Interactions of **ACMHCA** ligand and its metal complexes with VEGFER-2 enzyme and positioning main pocket. In comparison to the reference the original ligand and anticancer agent (*Cis-platin*).

Compound	2D receptor interaction	3D receptor interaction	3D Receptor Positioning
**Co-crystalized ligand**			
**ACMHCA ligand**			
**Ni (II)-ACMHCA1**			
**Co (II)-ACMHCA2**			
**Fe(III)-ACMHCA3**			
***Cis-platin***

i. The Co(II)-**ACMHCA 2** and Fe(III)-**ACMHCA 3** complexes interacted strongly with the receptor domain, while the Ni(II)-**ACMHCA 1** complex localized to a secondary pocket, accounting for its lower activity.
ii. The stability and docking scores of the complexes followed a trend aligned with in vitro results.
iii. The Fe(III)-**ACMHCA 3** complex emerged as the most promising complex with a docking score of -5.94 kcal/mol, stabilizing in the primary binding pocket *via* 6 distinct bonds with 4 crucial amino acids: GLU883, ASP1044, ILE1023, and HIS1024.
iv. The Co(II)- complex has a docking score of -5.83 kcal/mol, forming 5 critical bonds with GLU883 and ASP1044.
v. The Ni(II)- complex, with a lower docking score of -4.9 kcal/mol, bound to 3 non-crucial residues: ALA1063, SER923, and LYS1060.

Notably, the docking results are in strong agreement with the observed antitumor activities of the synthesized metal complexes. The Fe(III)-**ACMHCA** (**3**) complex displayed the highest activity against the HepG2 cell line (IC_50_ = 5.79 µg mL^−1^) and also achieved the strongest binding score (–5.94 kcal mol^−1^). The correlation indicates that higher antitumor activity (lower IC_50_) is associated with stronger binding affinity, which is supported by the positive linear relationship: IC_50_ = − 6.359 + 0.051 Docking score (kcal mol^−1^), *r* = *0.94*, *n* = 3.

Herein, although the docking scores of the synthesized complexes were more favorable than *cisplatin*, the in vitro cytotoxicity data did not surpass *cisplatin*, as shown by the IC₅₀ values (Fe–**ACMHCA**: 5.79 µg/mL *vs. cisplatin*: 3.58 µg/mL). This difference can be explained by several factors not captured by docking simulations, including solubility, cellular uptake, metabolic stability, and intracellular activation pathways, all of which strongly influence the overall cytotoxic response in living systems. Docking provides valuable insights into potential binding interactions at the molecular level, but it does not account for pharmacokinetic and physicochemical barriers that ultimately determine biological efficacy.

Moreover, chemical hardness (η) and softness (S) are global reactivity descriptors that can be linked to molecular docking studies to assess how a molecule’s reactivity affects its binding to a receptor. Higher hardness and lower softness values may indicate a compound’s increased likelihood of interacting with biological targets. This relationship is supported by linear regression analyses, which show a positive correlation between docking scores and hardness, and a negative correlation between docking scores and softness, as evidenced by the equations: Docking score = -9.502 + 2.430 η/eV, *r* = 0.92, *n* = 3 points and Docking score = -1.848–5.948 S/eV, *r* = 0.94, *n* = 3 points (Figs. S15 and S16).

The key conclusions indicate that Co(II)- and Fe(III) complexes showed high stability within the primary pocket, exhibited superior protein inhibition compared to **ACMHCA** and *cis platin*, whereas Ni(II)- complex resided in a secondary pocket, so displayed significantly lower activity. Moreover, docking data showed that Fe(III)-**ACMHCA** and Co(II)-**ACMHCA** complexes were highly stable within the primary pocket, whereas the Ni(II)-**ACMHCA** complex resided in a secondary pocket, correlating with its diminished efficacy.

## Conclusion

This study reports the successful synthesis of solvatochromic Ni(II), Co(II), and Fe(III) complexes of the tridentate semicarbazone ligand (**ACMHCA**). The semicarbazone ligand showed stronger fluorescence than its metal complexes, with higher excited-state dipole moments indicating enhanced solvent sensitivity. Structural optimization using DFT/TD-DFT correlated well with experimental data. The Fe(III)-**ACMHCA** complex exhibited the most promising biological activity, showing strong anticancer potential against HepG-2 cells (IC_50_ = 5.79 µg ml^− 1^) in line with its high docking score (-5.942 kcal/mol) inside the VEGFR-2 pocket. These findings highlight the Fe(III) complex as a prospective antimicrobial and anticancer agent, with potential implications for therapeutic development.

## Supplementary Information

Below is the link to the electronic supplementary material.

Supplementary Material 1

## Data Availability

Yes, availability of Data and Materials. The datasets used and/or analyzed during the current study are available from the corresponding author on reasonable request.
