# Supplementary material for: Design, biological evaluation, solvatochromic, DFT, and molecular docking studies of new metal complexes derived from a semicarbazone ligand
Source: Sci Rep. 2025 Nov 29;15:42856. doi: 10.1038/s41598-025-26629-2 (PMC12669734; doi:10.1038/s41598-025-26629-2)
Supplement: Supplementary file 1 — Supplementary Material 1 [file 41598_2025_26629_MOESM1_ESM.docx]

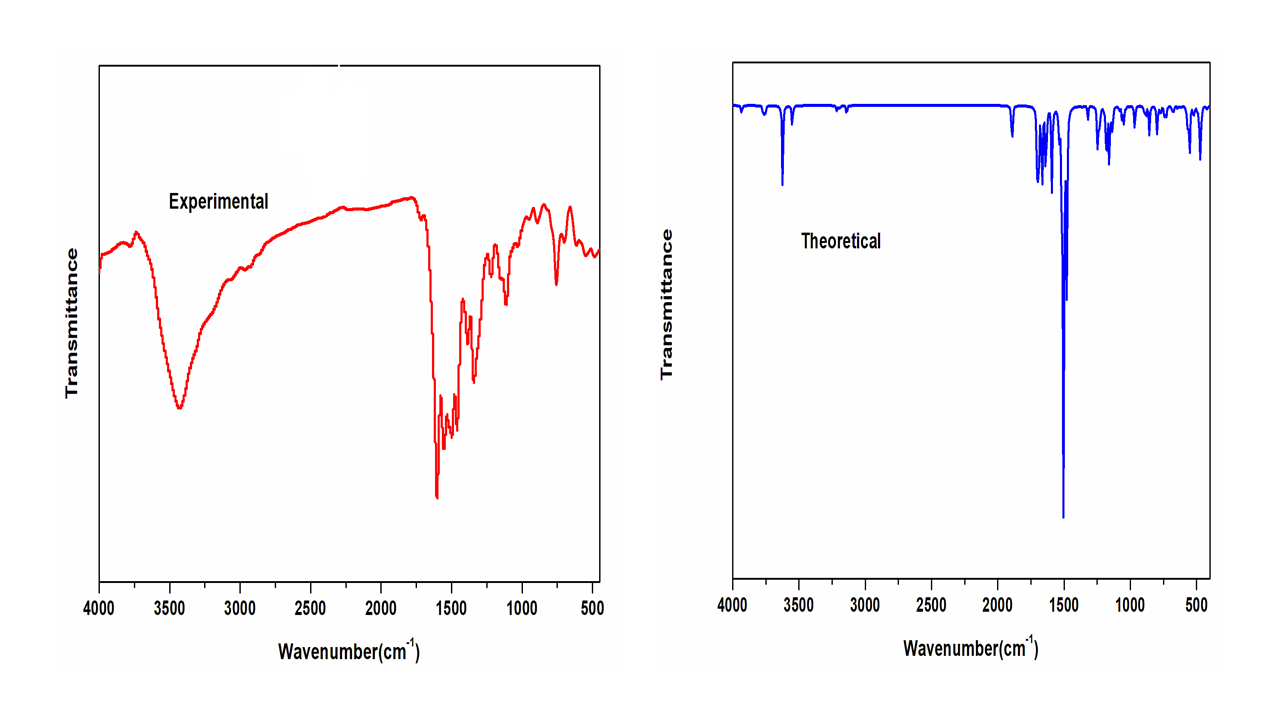


Fig. S1: Theoretical and experimental IR spectra of Ni(II)**-ACMHCA** complex **1**


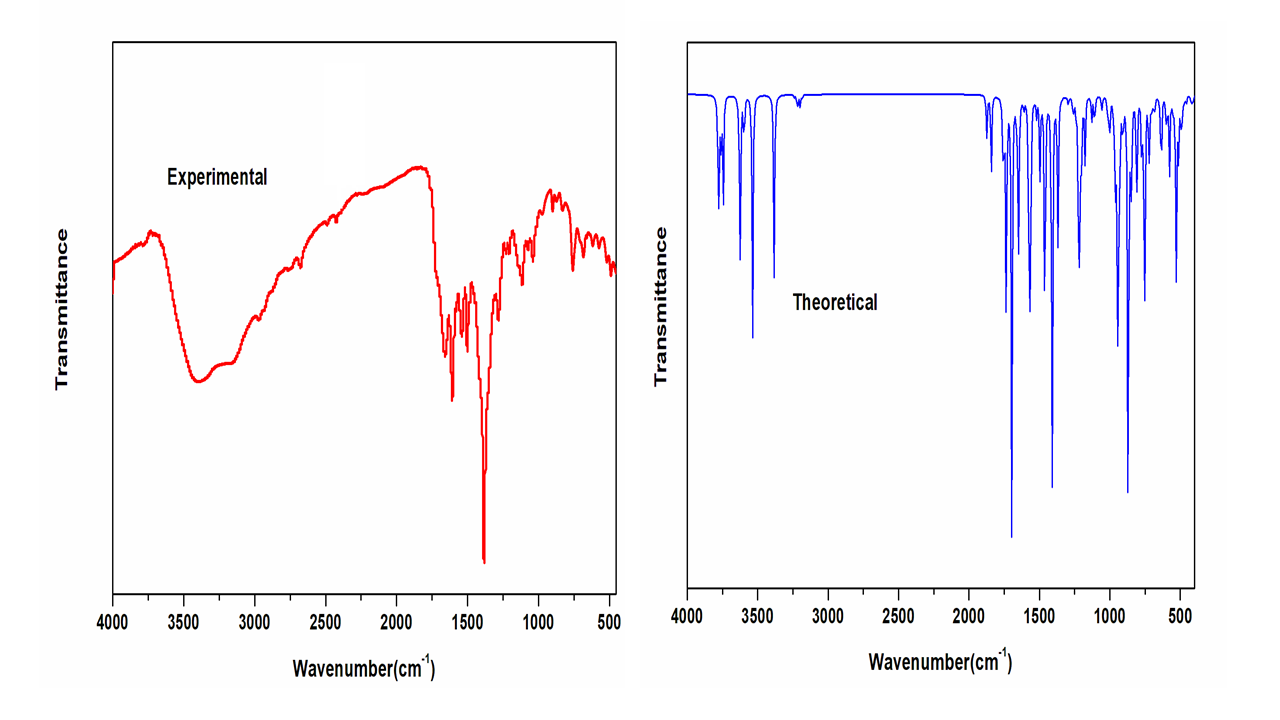


Fig.S2: Theoretical and experimental IR spectra of Fe(III)**-ACMHCA** complex **3**


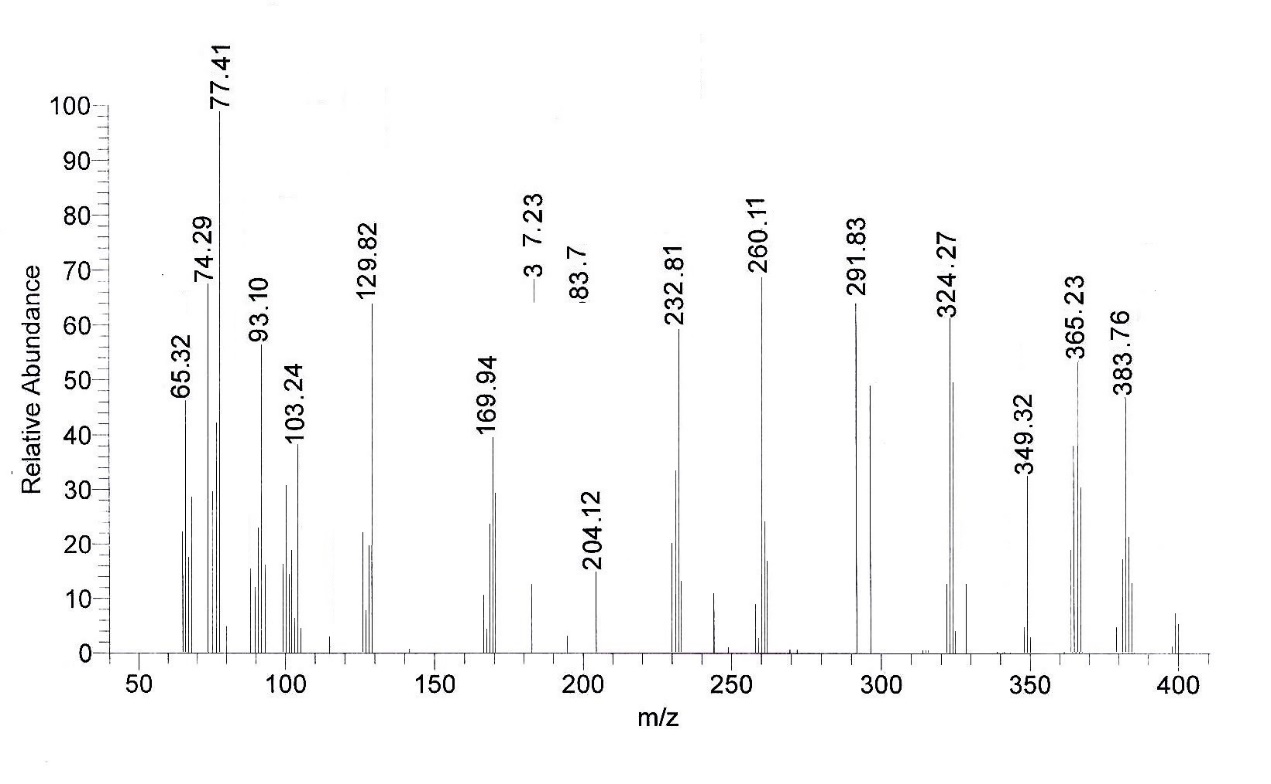


Fig. S3: Mass spectrum of Ni(II)-**ACMHCA** complex **1**


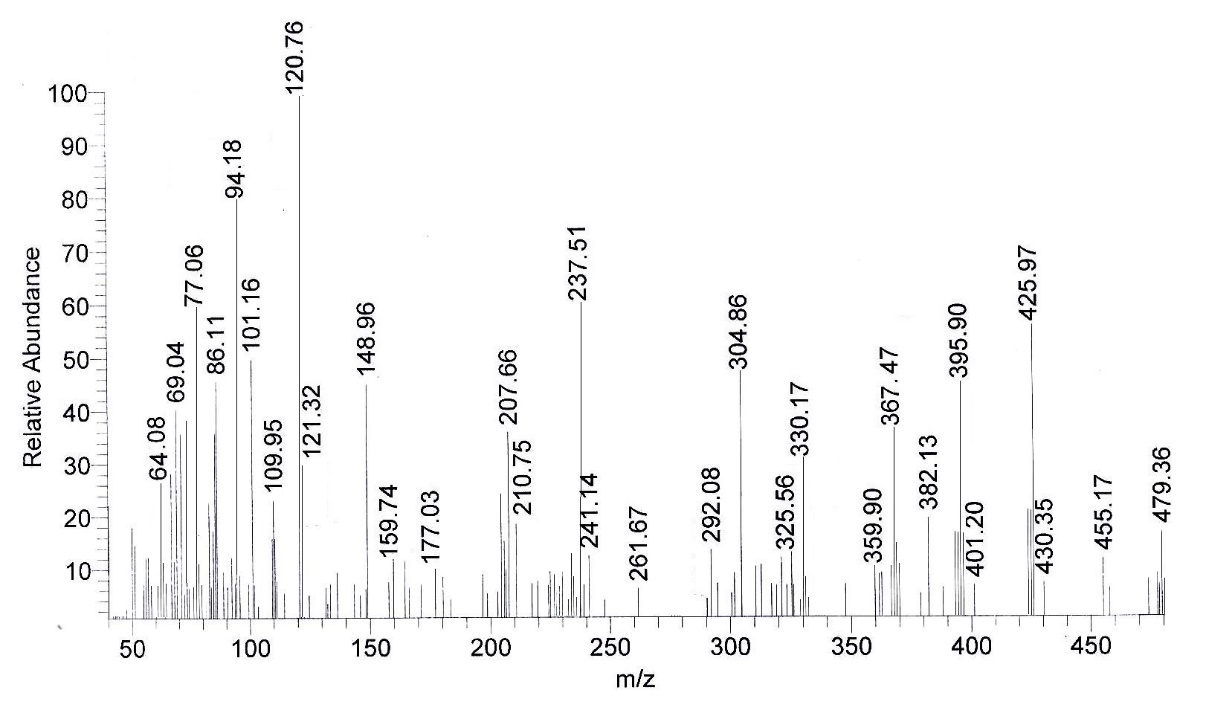


Fig. S4: Mass spectrum of Fe(III)-**ACMHCA** complex **3**


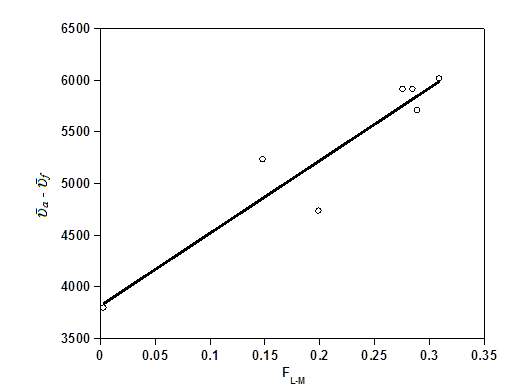


Fig. S5. The linear relationship of Stoke shift *versus* F_L-M_ for Fe(III)**-ACMHCA** complex **3**.


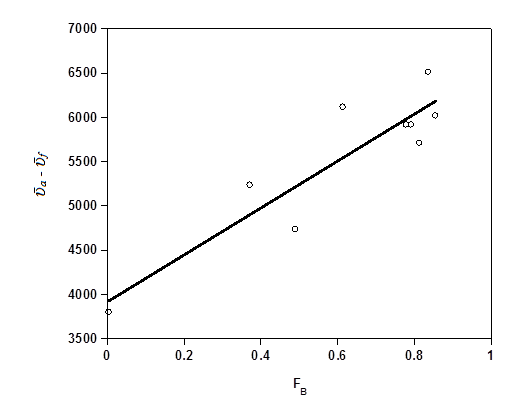


Fig.S6. The linear relationship of Stoke shift *versus* F_B_ for Fe(III)**-ACMHCA** complex **3**.


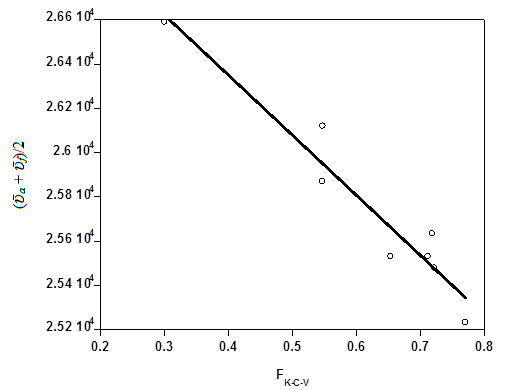


Fig.S7. The linear relationship between the average *versus* F_K-C-V_ for Fe(III)**-ACMHCA** complex **3**.


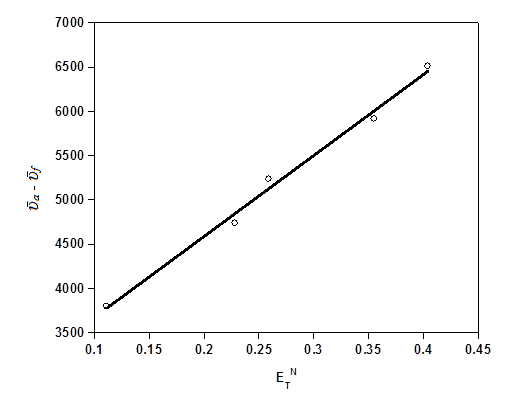


Fig. S8. The linear relationship between Stoke shift *versus* *E_T_^N^* for Fe(III)**-ACMHCA** complex **3**.


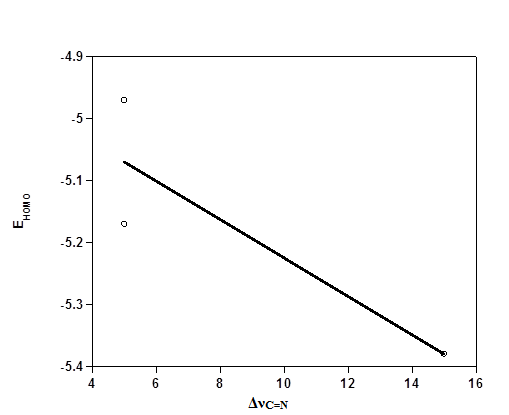


Fig. S9. The linear relationship between E_HOMO_ and Δν_C=N_.


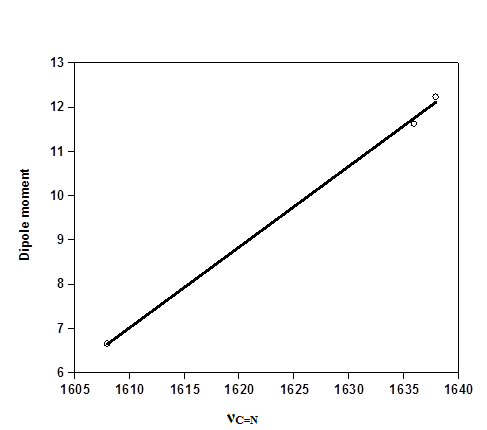


Fig. S10. The linear relationship between Dipole moment (µ) and ν_C=N_.

Fig. S11. The linear correlation of pIC_50_ *versus* E_LUMO_.

Fig.S12. The linear correlation of pIC_50_ *versus* hardness (η).

Fig.S13. The linear correlation of pIC_50_ *versus* Softness (S).

Fig.S14. The linear correlation of pIC_50_ *versus* dipole moment (µ).

Fig. S15. The linear correlation of docking score *versus* hardness (η).

Fig.S16. The linear correlation of docking score *versus* softness (S).

Table S1. Thermal gravimetric results of metal complexes.

| Complex | DTG peak (ºC ) | Temperature range (ºC) | Decomposition product lost (formula weight) | Weight loss (%) found (calculated) |
| --- | --- | --- | --- | --- |
| [(L)Ni(NO_3_)(H_2_O)].0.5CH_3_OH **1** | 70  184  424  Residue | 27-101  101-232  232-420  371-754 | -0.5CH_3_OH  -H_2_O  -HNO_3_+C_11_H_9_N_4_O  -NiO+C | 4.27(4.00)  4.53 (4.50)  70.30 (70.17)  20.50 (21.72) |
| [(L)Co(NO_3_)(H_2_O)].0.5CH_3_OH **2** | 73  294  402  Residue | 33-101  110-334  334-553 | - 0.5CH_3_OH  - HNO_3_+ H_2_O  - C_11_H_9_N_4_O_2_  -CoO | 4.35 (3.99)  21.47 (20.24)  56.83 (57.25)  17.35 (18.71) |
| [(L)Fe(H_2_O)_3_](NO_3_)_2_.0.5CH_3_OH **3** | 72  164  254  338  450  Residue | 53-80  80-200  200-311  311-356  356-550 | -0.5CH_3_OH  -3H_2_O+2HCN  -2NH_2_+O_2_  -2HNO_3_  -C_4_H_4_  _-_ FeO+5C | 3.44 (3.23)  21.05 (21.81)  13.70 (12.92)  24.56(25.44)  10.34 (10.49)  25.50 (26.65) |

Table S2. Calculated values of solvent parameters (ε, n), microscopic solvent polarity (E_T_^N^) and solvent polarity functions (F_1_, F_2_, F_3_), are recorded in increasing order ε.

| **Solvent** | ε | n | E_T_^N^ | F_1_(ε,n) | F_2_(ε,n) | F_3_(ε,n) |
| --- | --- | --- | --- | --- | --- | --- |
| 1,4 Dioxane | 2.25 | 1.4224 | 0.1636 | 0.0245 | 0.0499 | 0.290 |
| Benzene | 2.28 | 1.5010 | 0.1110 | 0.003 | 0.0040 | 0.300 |
| Chloroform | 4.81 | 1.4490 | 0.2593 | 0.1483 | 0.3709 | 0.825 |
| Ethyl acetate | 6.02 | 1.3724 | 0.2284 | 0.1996 | 0.4891 | 0.490 |
| THF | 7.58 | 1.407 | 0.228 | 0.210 | 0.549 | 0.551 |
| Isopropanol | 17.80 | 1.3993 | 0.5864 | 0.2641 | 0.7532 | 0.650 |
| Acetone | 20.70 | 1.3588 | 0.3548 | 0.2843 | 0.7903 | 0.654 |
| Ethanol | 24.50 | 1.3614 | 0.6543 | 0.2887 | 0.8127 | 0.924 |
| Methanol | 32.50 | 1.3284 | 0.7623 | 0.3086 | 0.8547 | 0.650 |
| DMF | 38.25 | 1.4300 | 0.4040 | 0.2750 | 0.8400 | 0.710 |

Table S3. Intercept (**C**), the slope (**S**), correlation coefficient (***r***) and number of data points (**n**) corresponding to statistical treatment of spectral shifts of metal complexes.

| Fe(III)-**ACMHCA** 3 | | | | | Co(II)-**ACMHCA** 2 | | | | Ni(II)-**ACMHCA** 1 | | | |  |
| --- | --- | --- | --- | --- | --- | --- | --- | --- | --- | --- | --- | --- | --- |
| n | ***r*** | **S** | **C** | **n** | | ***r*** | **S** | **C** | **n** | ***r*** | **S** | **C** |  |
| 7 | 0.94 | 7026.3 | 3818 | 6 | | 0.96 | 3658.3 | 4528.3 | 5 | 0.91 | 3509.8 | 4099.4 | **ν_stoke_ *vs* F_1_** |
| 9 | 0.91 | 2652.3 | 3914.5 | 8 | | 0.94 | 1960.9 | 4432.8 | 7 | 0.94 | 2805 | 3742.8 | **ν_stoke_ *vs* F_2_** |
| 7 | 0.97 | 2712.2 | 27435 | 8 | | 0.93 | 2001.5 | 27586 | 7 | 0.90 | 2484.5 | 27426 | **(ν_a_+ν_f_ )/2 *vs* F_3_** |
| 5 | 0.99 | 9140.4 | 2758.7 | 6 | | 0.88 | 7166.5 | 3288.8 | 5 | 0.97 | 8982.7 | 2964.7 | **ν_stoke_ *vs* E_T_^N^** |

Table S4. Solvent parameters analyzed by linear solvation energy relationships (LSFER).

| Solvent | DN^n^ | AN^n^ | E_T_^n^ | π* | β | α |
| --- | --- | --- | --- | --- | --- | --- |
| 1,4-Dioxane | 0.38 | 0.19 | 0.16 | 0.55 | 0.37 | 0.00 |
| Benzene | 0.00 | 0.15 | 0.11 | 0.59 | 0.10 | 0.00 |
| Chloroform | 0.10 | 0.42 | 0.26 | 0.58 | 0.10 | 0.20 |
| Ethyl acetate | 0.43 | 0.23 | 0.28 | 0.55 | 0.45 | 0.00 |
| Isopropanol | 0.93 | 0.61 | 0.55 | 0.48 | 0.84 | 0.76 |
| Acetone | 0.44 | 0.23 | 0.35 | 0.71 | 0.43 | 0.08 |
| Ethanol | 0.82 | 0.68 | 0.65 | 0.54 | 0.75 | 0.86 |
| Methanol | 0.77 | 0.75 | 0.76 | 0.60 | 0.66 | 0.98 |
| DMF | 0.69 | 0.29 | 0.41 | 0.88 | 0.69 | 0.00 |
| THF | 0.51 | 0.14 | 0.21 | 0.58 | 0.55 | 0.00 |

Table S5. Percentage of the linear solvation energy relationship's relative contribution (LSFER).

| Complex | % DN^n^ | % AN^n^ | % E_T_^n^ | % α | % β | % π* |
| --- | --- | --- | --- | --- | --- | --- |
| **1** | 35.32 | 12.83 | 1.00 | 11.16 | 34.20 | 5.47 |
| **2** | 22.41 | 6.13 | 16.85 | 12.39 | 17.10 | 25.11 |
| **3** | 40.22 | 1.72 | 8.28 | 0.023 | 38.39 | 11.37 |

Table S6. Quantum chemical descriptors for the studied compounds using B3LYP/GEN with 6-311G (d,p).

| µ, Ɗ | Chemical potential  Pi (eV) | Electrophilicity  ω (eV) | Electronegativity  χ (eV) | Hardness  Ƞ (eV) | Softness  S (eV^-1^) | E_gap_  (eV) | E_LUMO_  (eV) | E_HOMO_  (eV) | E_T_,  kcal/mol | Compound |
| --- | --- | --- | --- | --- | --- | --- | --- | --- | --- | --- |
| 5.84 | -3.25 | 2.56 | 3.25 | 2.07 | 0.48 | 4.14 | -1.18 | -5.32 | -5456.63 | **ACMHCA** |
| 11.62 | -3.63 | 3.64 | 3.63 | 1.81 | 0.55 | 3.08 | -2.09 | -5.17 | -2051.32 | **1** |
| 12.23 | -3.72 | 4.19 | 3.72 | 1.65 | 0.61 | 3.31 | -2.07 | -5.38 | -1971.84 | **2** |
| 6.65 | -3.56 | 4.49 | 3.56 | 1.41 | 0.71 | 2.82 | -2.15 | -4.97 | -1769.17 | **3** |

Table S7. The characteristic bond lengths and charge of **ACMHCA** ligand and its metal complexes.

| Compounds | Charges | | | |  | Bond length | | | | |
| --- | --- | --- | --- | --- | --- | --- | --- | --- | --- | --- |
|  | O_14_ | O_27_ | N_21_  C=N coord. | M | C_4_=O_14_ | C_23_-O_27_ | M-O_14_ | M-O_27_ | M-N_21_ | C_15_=N_21_  Coord. |
| **ACMHCA** | -0.359 | -0.372 | -0.283 | ----- | 1.230 | 1.211 | ----- | ----- | ----- | 1.285 |
| **1** | -0.562 | -0.623 | -0.389 | 0.935 | 1.258 | 1.306 | 1.990 | 1.907 | 2.009 | 1.291 |
| **2** | -0.424 | -0.499 | -0.294 | 0.590 | 1.249 | 1.260 | 2.047 | 2.176 | 2.123 | 1.329 |
| **3** | -0.474 | -0.463 | -0.289 | 0.245 | 1.268 | 1.265 | 2.117 | 2.335 | 1.876 | 1.296 |

Table S8. The dipole moment (μ), the mean polarizability (α), and the anisotropy of the polarizability (∆α) for the prepared complexes.

| Complex | µ_x_ | µ_y_ | µ_z_ | μ_total_ | α_xx_ | α_yy_ | α_zz_ | α_xy_ | α_xz_ | α_yz_ | <α> (au) | <α> (esu)  x10^-23^ | ∆α (au) | ∆α (esu)  x 10^-23^ |
| --- | --- | --- | --- | --- | --- | --- | --- | --- | --- | --- | --- | --- | --- | --- |
| **1** | 6.08 | 8.50 | 5.08 | 11.62 | 110.81 | 138.13 | 140.15 | -9.64 | -11.17 | -13.58 | 129.69 | 1.92 | 37.12 | 5.50 |
| **2** | 3.00 | 11.13 | 4.05 | 12.23 | 91.34 | 130.29 | 151.53 | -7.14 | -2.09 | -25.24 | 124.39 | 1.84 | 53.01 | 7.85 |
| **3** | 5.57 | 1.00 | -3.47 | 6.64 | 112.90 | 116.01 | 139.98 | 11.63 | 3.53 | -3.83 | 122.97 | 1.82 | 32.85 | 4.86 |

Table S9. The calculated hyperpolarizability (β_tot_) components for the synthesized complexes.

| Complex | β_xxx_ | β_xyy_ | β_xzz_ | β_yyy_ | β_yxx_ | β_yzz_ | β_zzz_ | β_zyy_ | β_total_ (au) | β_total_ (esu)  x 10^-30^ |
| --- | --- | --- | --- | --- | --- | --- | --- | --- | --- | --- |
| **1** | -77.22 | 51.63 | -51.17 | 102.02 | 34.10 | 21.17 | 93.22 | -0.64 | 198.29 | 1.71 |
| **2** | -149.16 | 8.81 | -45.57 | 120.53 | 39.04 | 3.92 | 53.85 | 4.67 | 254.40 | 2.19 |
| **3** | -23.38 | 2.45 | -58.01 | 39.26 | -64.11 | -2.67 | 2.79 | -2.67 | 85.95 | 0.74 |

Table S10: Comparative analysis for Interactions of **ACMHCA** and its analogues metal complexes with VEGFER-2 Enzyme sites, Interaction type, Distances, Binding scores and Energies main pocket, in comparison to the reference the original ligand and anticancer agent (*Cis-platin*).

| **Compound** | **Ligand** | **Receptor** | **Type** | **Distance (Å)** | **Energy (kcal/mol)** | **Receptors** | **Docking Score** **(kcal/mol)** |
| --- | --- | --- | --- | --- | --- | --- | --- |
| **Co-crystalized ligand** | N12 15  N15 19  N31 53  O14 18  N28 56  5-ring  6-ring | OE2  OE2  O  N  N  CG1  CB | H-donor  H-donor  H-donor  H-acceptor  H-acceptor  pi-H  pi-H | 2.83  2.85  2.84  3.07  2.98  4.35  3.55 | - 4.9  - 3.8  - 3.3  - 2.4  - 5.0  - 0.6  - 0.5 | GLU 883  GLU 883  GLU 915  ASP 1044  CYS 917  VAL 846  ASP 1044 | - 6.96 |
| **ACMHCA ligand** | N14 21  N16 24 | OE2  OE2 | H-donor  H-donor | 2.87  3.16 | - 7.0  - 1.8 | GLU 883  GLU 883 | - 5.45 |
| **Ni(II)-ACMHCA**  **1** | N11 15  O20 29  6-ring | O  OG  CB | H-donor  H-acceptor  pi-H | 2.87  2.78  4.12 | - 1.9  - 11.4  - 0.6 | ALA 1063  SER 923  LYS 1060 | - 4.9 |
| **Co(II)-ACMHCA**  **2** | N11 15  O24 33  N11 15  N11 15  6-ring | OE2  OD2  OE1  OE2  CB | H-donor  H-donor  Ionic  Ionic  pi-H | 3.16  2.81  3.89  3.16  3.59 | - 1.9  - 2.6  - 0.7  - 3.5  - 0.7 | GLU 883  ASP 1044  GLU 883  GLU 883  ASP 1044 | - 5.83 |
| **Fe(III)-ACMHCA**  **3** | N11 15  O20 30  O21 33  O22 36  N11 15  6-ring | OE2  OD2  O  O  OE2  CB | H-donor  H-donor  H-donor  H-donor  Ionic  pi-H | 3.45  2.61  2.65  3.08  3.45  3.63 | - 1.0  - 0.9  - 0.9  - 0.9  - 2.1  - 0.8 | GLU 883  ASP 1044  ILE 1023  HIS 1024  GLU 883  ASP 1044 | - 5.94 |
| ***Cis-platin*** | CL4 10  CL5 11 | N  N | H-acceptor  H-acceptor | 3.92  3.04 | - 0.7  - 1.6 | ASP 1044  ASP 1044 | - 4.19 |

Table S11: Illustration of 3D Receptor Positioning of **ACMHCA** ligand its metal complexes with VEGFER-2 Enzyme main pocket.

| **Co-crystalized ligand 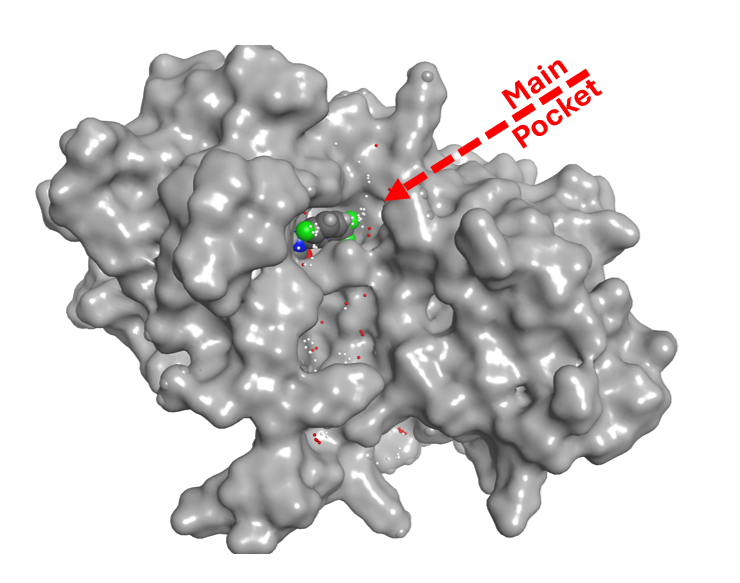** | ***Cis-Platin***  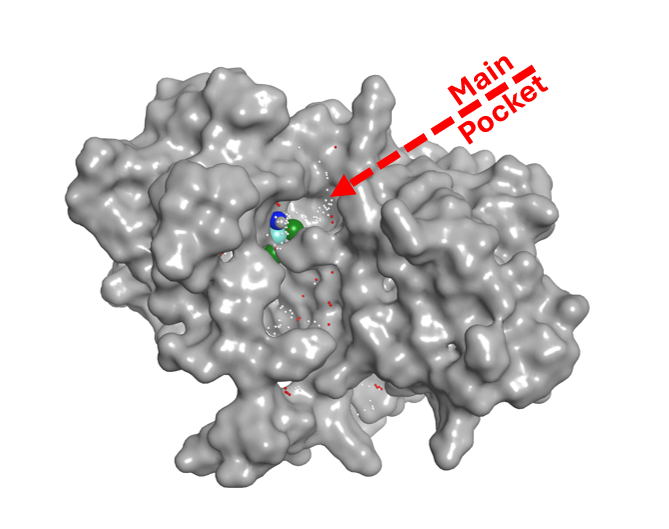 |
| --- | --- |
| **ACMHCA ligand** 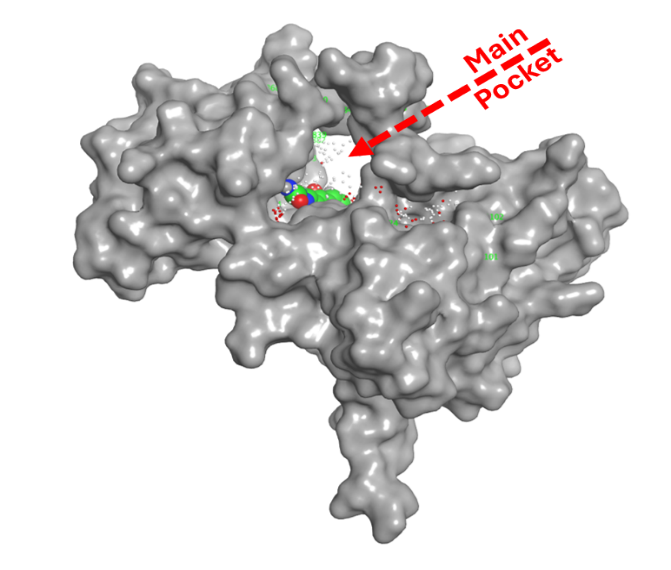 | **Ni-ACMHCA complex 1**  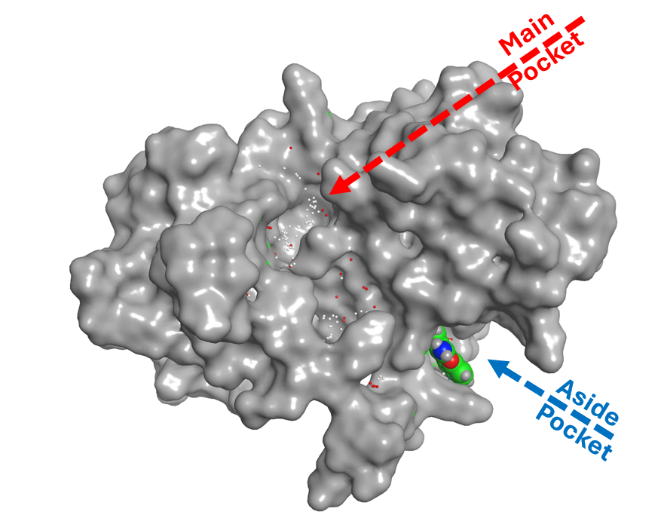 |
| **Co-ACMHCA complex 2**  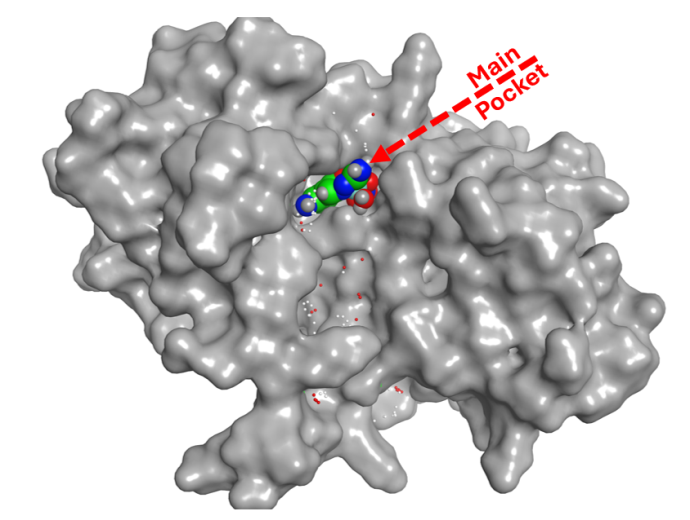 | **Fe-ACMHCA complex 3**  **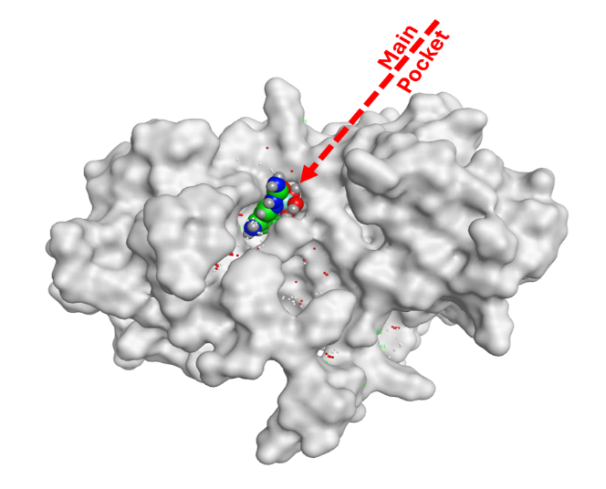** |

***2.2. Measurements***

Elemental analyses (C, H and N) were carried out using Vario El-Elementar at the Ministry of Defense, Chemical War Department. Analysis of the metal ion followed the decomposition of an accurate weight of metal complexes with concentrated HNO_3_, neutralizing with ammonia and titrating with EDTA Decomposition temperatures of the compound were determined using a Stuart *SMP3* melting point apparatus. IR spectra were recorded by FT IR Nicolet IS10 spectrometer. Utilizing a Jasco UV-vis spectrophotometer (V-550), the spectra (200–800 nm) were measured as Nujol mulls or DMF solutions. The fluorescence spectrum data of the compounds was acquired at Ain Shams University in Cairo, Egypt using a Perkin Elmer LS 55 Luminescence Spectrometer (USA). Molar conductivity measurements of 10^-3^ M solutions of the metal complexes in DMF were dignified on the Corning conductivity meter NY 14831 model 441. Magnetic susceptibilities of metal complexes were measured by Gouy method at room temperature using Shelwood Scientific, Cambridge Science Park, magnetic susceptibility balance (England). The effective magnetic moments were calculated using the relation μ_eff_ = 2.828(cm.T)^1/2^B.M., where cm is the molar susceptibility corrected using Pascal's constants for the diamagnetism of all atoms in compounds. TGA measurements were carried out from room temperature up to 800 ^o^C at a heating rate of 10 ^o^C/min on a Shimadzu-50 thermal analyzer.
